# Author Correction: The impact of rare germline variants on human somatic mutation processes

**DOI:** 10.1038/s41467-023-41324-4

**Published:** 2023-09-06

**Authors:** Mischan Vali-Pour, Solip Park, Jose Espinosa-Carrasco, Daniel Ortiz-Martínez, Ben Lehner, Fran Supek

**Affiliations:** 1 Centre for Genomic Regulation (CRG), The Barcelona Institute of Science and Technology (BIST), Barcelona, Spain; 2 Universitat Pompeu Fabra (UPF), Barcelona, Spain; 3 Centro Nacional de Investigaciones Oncológicas (CNIO), Madrid, Spain; 4 Institute for Research in Biomedicine (IRB Barcelona), The Barcelona Institute of Science and Technology (BIST), Barcelona, Spain; 5 Catalan Institution for Research and Advanced Studies (ICREA), Barcelona, Spain

**Keywords:** Cancer genomics, Rare variants, Computational biology and bioinformatics, Cancer genetics

Correction to: *Nature Communications* 10.1038/s41467-022-31483-1, published online 28 June 2022

The original version of this Article omitted from the author list the following authors: the 2nd author Solip Park, who is from the ‘Centro Nacional de Investigaciones Oncológicas (CNIO), Madrid, Spain’; the 3rd author Jose Espinosa-Carrasco, who is from the ‘Institute for Research in Biomedicine (IRB Barcelona), The Barcelona Institute of Science and Technology (BIST), Barcelona, Spain’; and the 4th author Daniel Ortiz-Martínez, who is from the ‘Institute for Research in Biomedicine (IRB Barcelona), The Barcelona Institute of Science and Technology (BIST), Barcelona, Spain’. The corrected version of the Acknowledgements also removes the following from the original version: ‘We thank Solip Park for method consultations, comments, and discussions.’

Additionally, the Author Contributions statement has been corrected as follows: ‘M.V.P. collected and curated the data, implemented software, performed all analyses, visualized the data, and interpreted the results. S.P. supervised the processing of common germline variants for the PCA analysis and the definition of rare germline variants. J.E.C. performed data retrieval and bioinformatics preprocessing of the germline data of a part of the genomic data analyzed (TCGA WES data sets BRCA, COAD, GBM, KICH, KIRC, KIRP, LIHC, PAAD, READ, STAD, THCA and UCEC). D.O.M. performed data retrieval and bioinformatics preprocessing of a part of the genomic data analyzed (ICGC WGS data sets ESAD-UK and MELA-AU). F.S. and B.L. conceptualized the analysis, devised the methodology, interpreted results and supervised the study. M.V.P., B.L. and F.S. drafted the manuscript jointly.’

This has been corrected in both the PDF and HTML versions of the Article.

