# Supplementary material for: The impact of rare germline variants on human somatic mutation processes
Source: Nat Commun. 2022 Jun 28;13:3724. doi: 10.1038/s41467-022-31483-1 (PMC9240060; doi:10.1038/s41467-022-31483-1)
Supplement: Supplementary file 1 — Supplementary Information [file 41467_2022_31483_MOESM1_ESM.pdf]

Supplementary Information

The impact of rare germline variants on human somatic mutation processes

Mischan Vali Pour, Ben Lehner, Fran Supek

Contents

List of Figures III

List of Tables IV

1 Supplementary Note 1

1.1 *SETD2* associating in colorectal cancer with dMMR$_{VAE1}$ 1
1.2 Genes associating with a somatic feature enriched in brain and liver cancer 1
1.3 Inflation analysis 1

2 Supplementary Figures 4

2.1 Inflation Analysis 4
2.2 Estimation of False Discovery Rates 5
2.3 Power Analysis 6
2.4 Subsampling 8
2.5 Overview of Discovered Hits and/or Replicated Hits 12
2.6 Distributions of Rare Variants 16
2.7 Network Analysis 19
2.8 Somatic Input Features 21
2.9 Extraction of Independent Components 23
2.10 Extracting Components using Variational Autoencoders 27
2.11 Overview of 29 Extracted Components 32
2.12 Correlation between IC9 (Sig.11+19) and telomere features 34
2.13 Sample Level Quality Control and Extraction of Individuals of European Ancestry from Common Germline Variants 35
2.14 Comparison Between WES and WGS Extracted Somatic Features . . . 40

3 Supplementary Tables 42

3.1 Replicates Genes - Minimal Distances 42
3.2 Genomic Regions 43
3.3 Somatic Components 44
3.4 Cancer Types 45

4 Supplementary References 51

List of Figures

1 Inflation analysis 4
2 Estimation of false discovery rates 5
3 Power Analysis 6
4 Lower-bound of % of variance explained for different known high effect size genes 7
5 Expected number of replicated hits at FDR 1 % hits 8
6 Subsampling of validation cohort 8
7 Number of replicated associations between *BRCA1* and dHR_ICA in pan-cancer in subsampling analysis 9
8 Number of replicated associations between *BRCA2* and dHR_ICA in pan-cancer in subsampling analysis 9
9 Number of replicated associations between *PALB2* and dHR_ICA in pan-cancer in subsampling analysis 10
10 Number of replicated associations between *MLH1* and dMMR_ICA in pan-cancer in subsampling analysis 10
11 Number of replicated associations between *MSH2* and dMMR_VAE1 in pan-cancer in subsampling analysis 11
12 Overview of number of discovered and validated hits 12
13 Replicated hits at a FDR of 2 % 13
14 Number of individuals with rare pLOF variants (and somatic LOH) in genes replicating at a FDR of 2 % 14
15 Seven genes associated with a somatic component in $\geq$ 1 cancer type . 15
16 Overall occurrence of pLOF variants and pLOF variants + somatic LOH in the cohorts 16
17 Number of individuals carrying a rare pLOF variant in one of the genes replicating at a FDR of 2 % 17
18 Frequency of pLOF variants in analyzed cancer dataset vs. control samples in gnomAD v2.1 18
19 Network analysis (HumanNet) supports the role of rare germline variation in somatic mutational processes 19
20 Gene prioritization based on protein interactions with known dHR/dMMR genes within network 20
21 Gene prioritization based on strength of protein interactions to neighbours within network 20
22 Distribution of all 56 somatic features in TCGA-WES 21
23 Distribution of all 56 somatic features in PCAWG_Hartwig-WGS 22
24 Finding the optimal number of independent components 23

25 Selection of 15 independent components for further analysis 24
26 Pearson correlations between all 30 independent components which were extracted using 15 components and k-medoid clustering with k = 30 24
27 Overview of strongest contributing features to the independent components 25
28 Several independent component scores were enriched in specific tissue of origins 26
29 Finding the optimal hyperparameters for the variational autoencoder . . 27
30 Correlation with biologically relecant components increased with increasing number of components extractions and quickly reached saturation 28
31 Number of hidden layers barely made a difference on the extracted components in the latent space of the variational autoencoder 28
32 Some VAE-derived components were not captured in the independent component analysis 29
33 Overview of strongest contributing features to the variational autoencoder derived components 30
34 Several VAE-derived component scores were enriched in specific tissue of origins 31
35 Distribution of all 29 somatic components in TCGA -WES 32
36 Distribution of all 29 somatic components in PCAWG_Hartwig -WGS . . 33
37 Pearson correlation between IC9 (Sig.11+19) and telomere features . . 34
38 Identification of individuals with outlying total number of variants, outlying heterozygosity rate or high relatedness in TCGA -WES. 35
39 Identification of individuals with outlying total number of variants, outlying heterozygosity rate or high relatedness in PCAWG_Hartwig -WGS . . . 36
40 Principal component analysis on common germline variants in TCGA - WES 37
41 Principal component analysis on common germline variants in PCAWG_Hartwig -WGS 38
42 Extraction of European individuals in TCGA -WES 39
43 Extraction of European individuals in PCAWG_Hartwig -WGS 40
44 Comparison between WES and WGS extracted somatic features in TCGA 41

List of Tables

1 Closest distance between newly replicated genes at a FDR of 2 % and known dMMR/dHR genes 42

2 Covered Genomic Regions with WES and WGS Masks 43
3 Somatic Component Names 44
4 TCGA Study Abbreviation 45
5 PCAWG Study Abbreviation 46
6 Cancer Type Names 46
7 Overview of sample sizes 47
8 Matching GTEx tissues with cancer types in TCGA for PEXT 48
9 Matching GTEx tissues with cancer types in PCAWG for PEXT 49
10 Matching GTEx tissues with cancer types in Hartwig for PEXT 50

1 Supplementary Note

1.1 *SETD2* associating in colorectal cancer with dMMR$_{VAE1}$

There is considerable support for the association of *SETD2* with dMMR from previous studies:

1. Biochemistry: the SETD2 protein deposits the histone mark H3K36me3, which can recruit the MSH6 subunit of the MMR complex MutSa to chromatin[8].
2. Experiments on human cell lines show that removing the SETD2 protein can cause microsatellite instability[8] and that it changes the distributions of mutation rates with respect to H3K36me-marked regions[9].
3. In human cancer data tumours with mutations in *SETD2* (double deletion or somatic mutation) have a dMMR-associated mutation phenotype[10].

In our opinion, these prior studies would be very strong evidence to support this dMMR association in *SETD2* germline variants as well.

1.2 Genes associating with a somatic feature enriched in brain and liver cancer

Beyond the dHR and dMMR-related components, the component associated with the largest number of genes was component Sig.11+19, which is enriched for SNV signatures RefSig 11 and 19[1] (Fig. 2d). This component is enriched in brain and liver cancers (Supplementary Fig. 37). Signature 11 has been reported to be enriched in brain cancers, associated with temozolomide treatment[2], and is similar to the signature which results from the treatment with the DNA methylating agent 1,2-Dimethylhydrazine[3]. The cause of signature 19 is unknown and it has been mostly identified in brain, liver and blood cancers[1]. At a FDR of 1 %, the genes ASCC2, FANCC, NCAPG2 and POT1 associated with this component in the pan-cancer analysis, as do NUDT7, PIF1, and SOS1 at a more permissive 2 % FDR. POT1 and PIF1 interact with each other[4] (Extended Data Fig. 6e) and both have functions in telomere maintenance[5,6], but we did not detect any correlation between this component and reported telomere features[7] (Supplementary Fig. 37).

1.3 Inflation analysis

The tests showed little evidence of inflation when considering models in which at least 100 genes were tested. Overall there was slight deflation (median: 0.78; 1st quartile:

0.55; 3rd quartile: 0.97; max: 2.27) (Supplementary Fig. 1), suggesting conservatively biased test results. Inflated cases were discarded (cut-off at lambda $\geq$ 1.5; 19 out of 1,909 discarded).

2 Supplementary Figures

2.1 Inflation Analysis

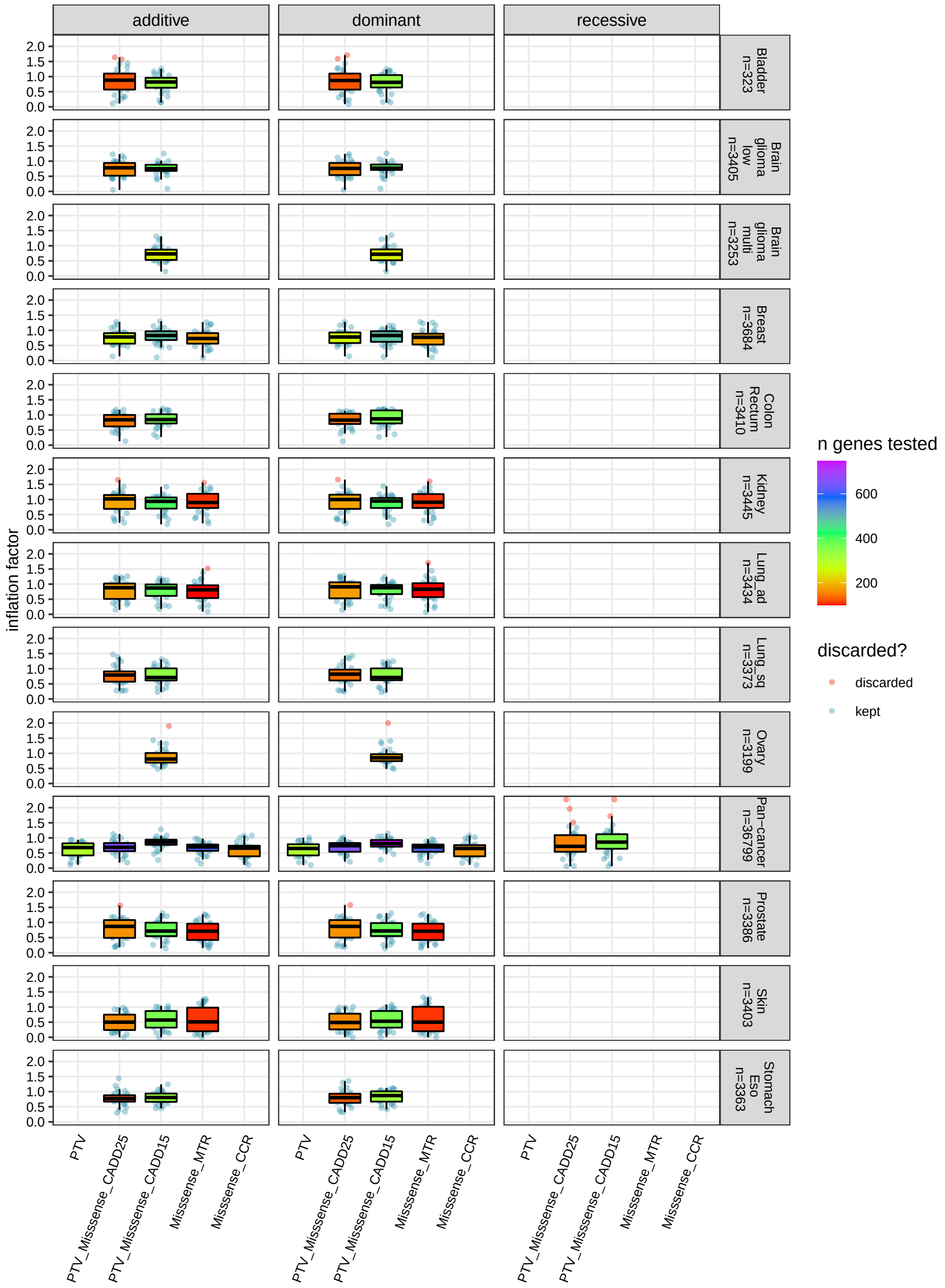

Supplementary Fig. 1: Inflation analysis. Overview of inflation factors (y-axis) across rare pLoF variant sets (x-axis), across cancer types (rows), and across models of inheritance (columns). Color code for box plots illustrates the number of tested genes for the respective scenario. Inflation factors were only calculated when at least 100 genes were tested, and inflation factors $\geq$ 1.5 were discarded (red point). Each data point represents the calculated inflation factor for one somatic component. Number of individuals in each cancer type shown in subtitle of each plot. Centre of each boxplot shows median value, bounds of box at 25th and 75th percentiles and minimum and maximum extend to the smallest and largest value, but not more than 1.5 times the interquartile range.

2.2 Estimation of False Discovery Rates

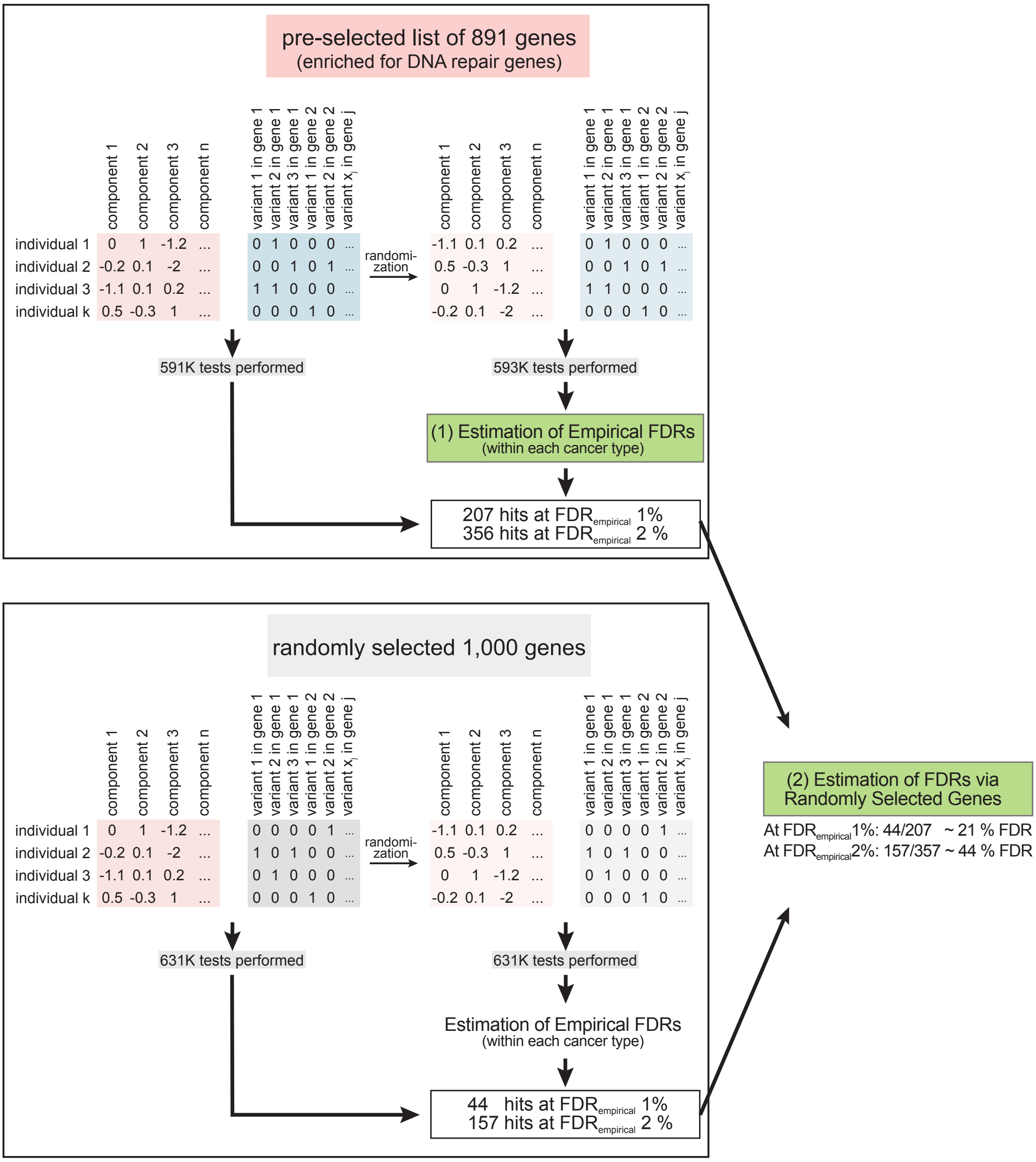

Supplementary Fig. 2: Estimation of false discovery rates. Schematic illustration of the approach. Firstly, testing was performed using the pre-selected 891 genes. Then, randomization was performed shuffling the rows within cancer types, effectively breaking down the link between individuals and somatic components. Testing was performed with the randomized somatic component matrix as well and empirical FDRs were calculated based on the randomization for each cancer type (top half of plot). The same approach was repeated with a random set of 1,000 genes after excluding the pre-selected gene list and any gene interacting with a gene from the pre-selected gene list (bottom half of plot). The number of genes replicating via the randomly selected list of genes at a specific FDR was divided by the number of genes replicating with the pre-selected list to get a conservative FDR estimate.

2.3 Power Analysis

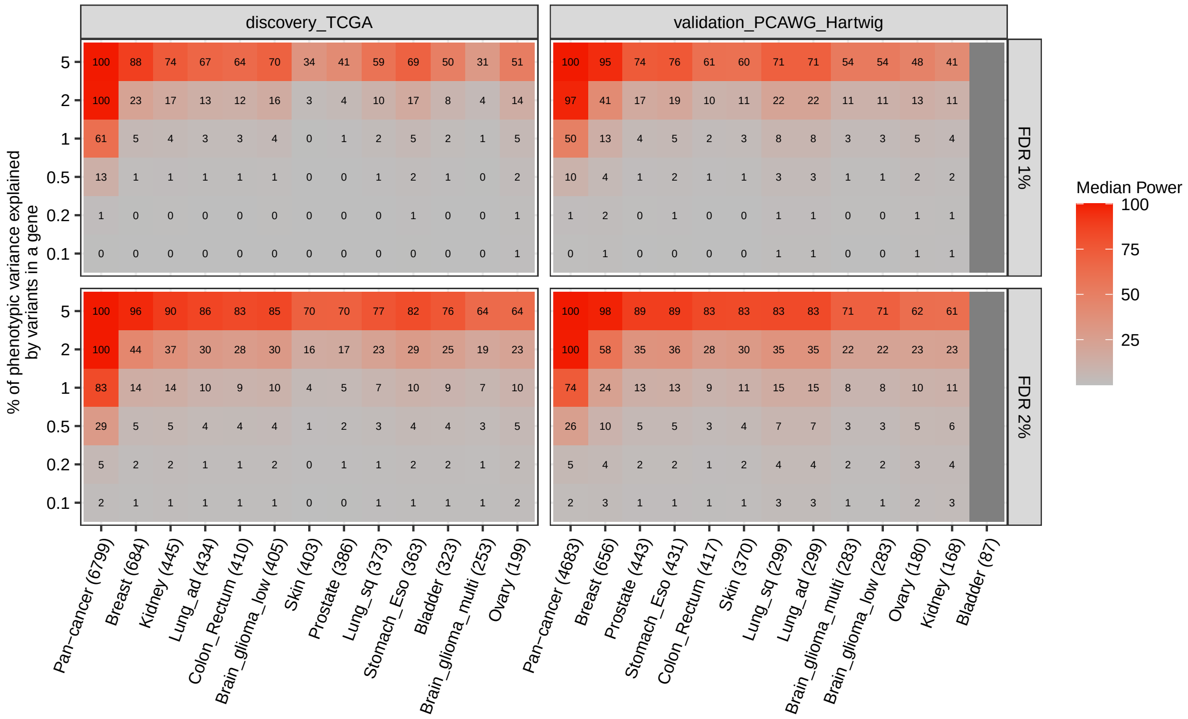

Supplementary Fig. 3: Power Analysis. Power analysis was performed with PAGEANT[11] for SKAT. Statistical power depending on the sample size (shown by different cancer types and pan-cancer on x-axis), % of phenotypic variance explained by variants in a gene (y-axis), and threshold for calling significance (1st row for 1 % FDR and 2nd row for 2 % FDR). Median of statistical power shown by color code and rounded numbers in each tile.

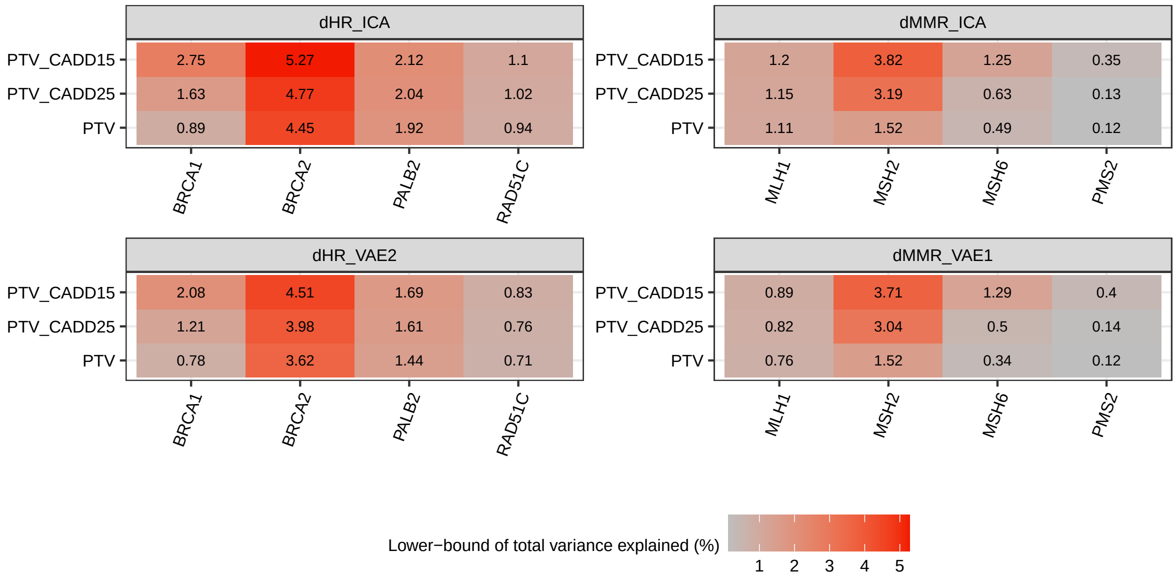

Supplementary Fig. 4: Lower-bound of % of variance explained for different known high effect size genes. Total variance explained (R2) was estimated in a multiple linear regression (see Methods) for different gene-phenotype-variant set combinations using the dominant model in the pan-cancer analysis on the discovery cohort. Estimates can be used as reference points for interpreting the power analysis.

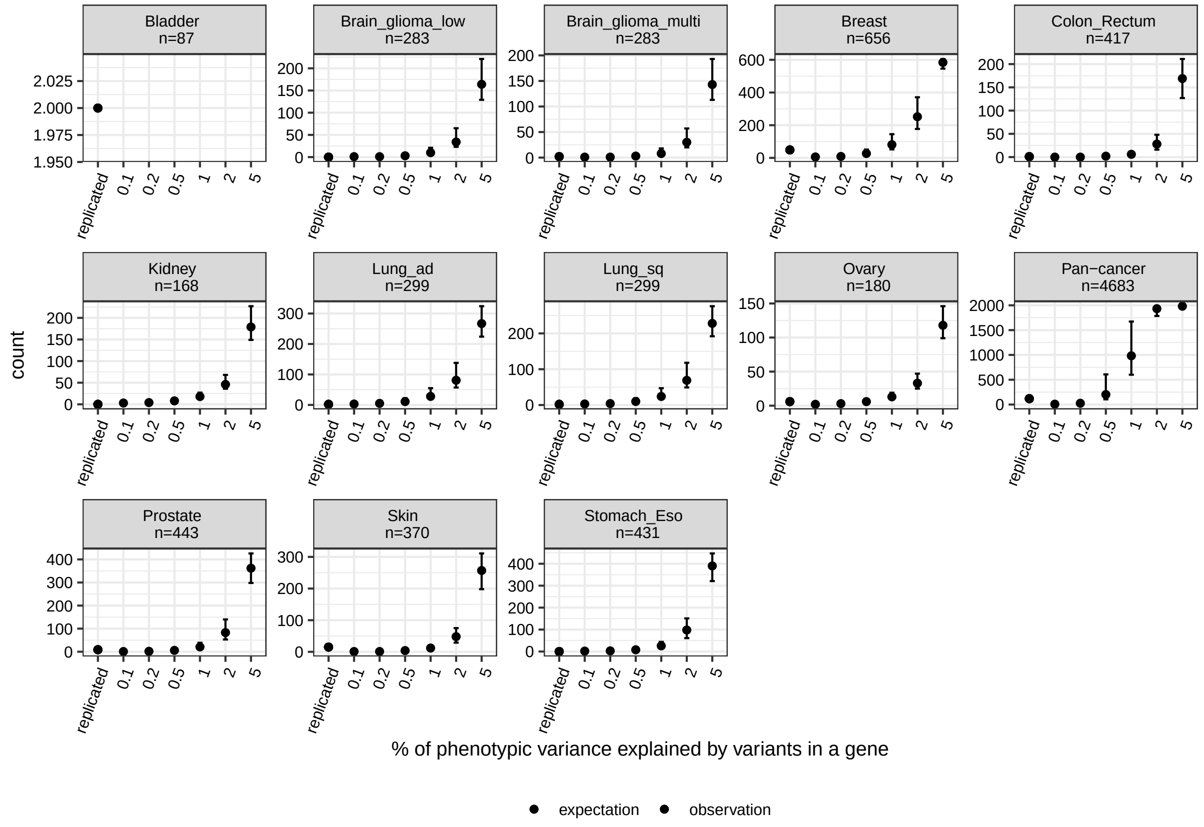

Supplementary Fig. 5: Expected number of replicated hits at FDR 1 % hits. Shown number of replicated hits in the validation cohort at a FDR of 1 % (red bar) in each cancer type and pan-cancer. Data points show expected number of replicated hits based on the estimated median statistical power for different %es of explained variance by variants in a gene. Error bars reflect the expected numbers based on the 25th and 75th quantile of the estimated statistical power with PAGEANT[11]. Number of individuals in each cancer type shown in subtitle of each plot.

2.4 Subsampling

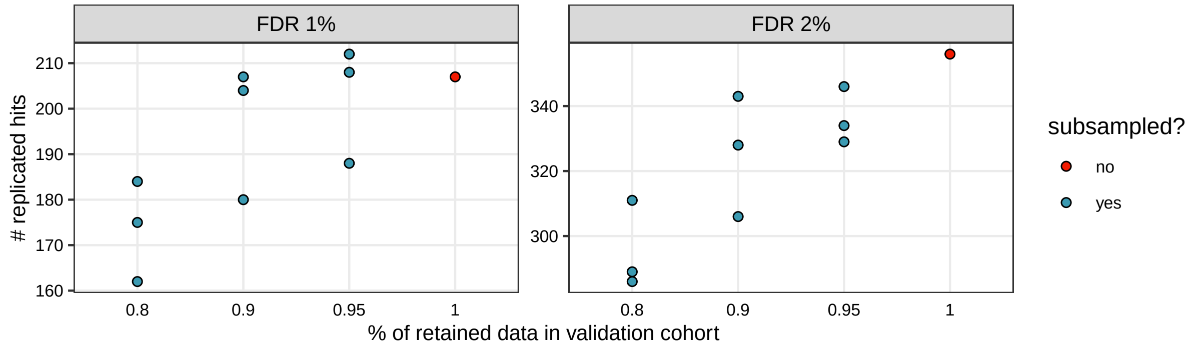

Supplementary Fig. 6: Subsampling of validation cohort.

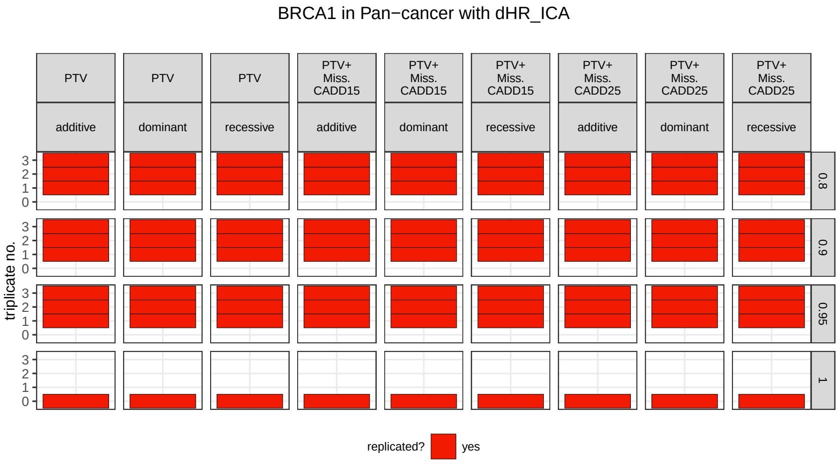

Supplementary Fig. 7: Number of replicated associations between *BRCA1* and dHR_ICA in pan-cancer in subsampling analysis. Showing replicated hits (red tile) for each pLoF rare variant set and model of inheritance combination (columns) for each subsampling (rows). Data of the validation cohort was randomly subsampled retaining 95, 90, and 80 % of the data three times for each subsampling (triplicate number on y-axis). Bottom row showing associations without any subsampling (100 % of the data).

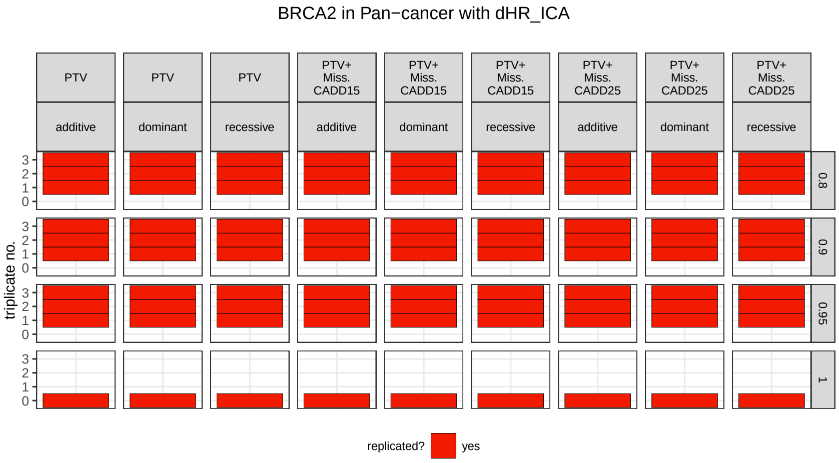

Supplementary Fig. 8: Number of replicated associations between *BRCA2* and dHR_ICA in pan-cancer in subsampling analysis. Showing replicated hits (red tile) for each pLoF rare variant set and model of inheritance combination (columns) for each subsampling (rows). Data of the validation cohort was randomly subsampled retaining 95, 90, and 80 % of the data three times for each subsampling (triplicate number on y-axis). Bottom row showing associations without any subsampling (100 % of the data).

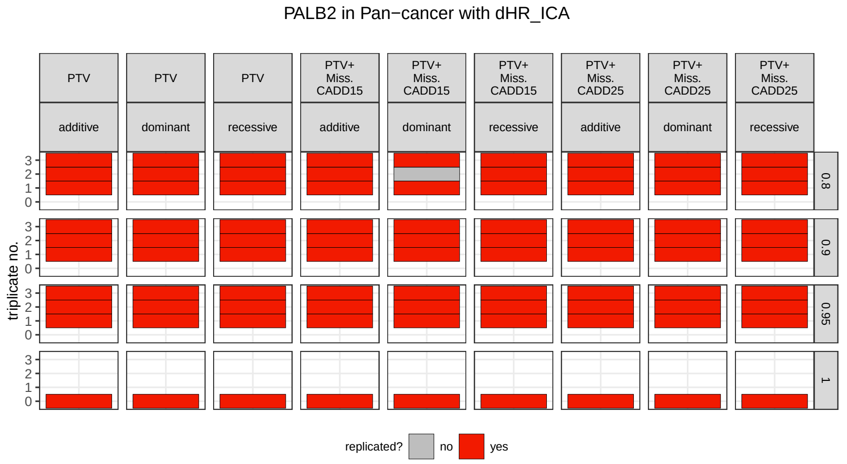

Supplementary Fig. 9: Number of replicated associations between *PALB2* and dHR_ICA in pan-cancer in subsampling analysis. Showing replicated hits (red tile) for each pLoF rare variant set and model of inheritance combination (columns) for each subsampling (rows). Data of the validation cohort was randomly subsampled retaining 95, 90, and 80 % of the data three times for each subsampling (triplicate number on y-axis). Bottom row showing associations without any subsampling (100 % of the data).

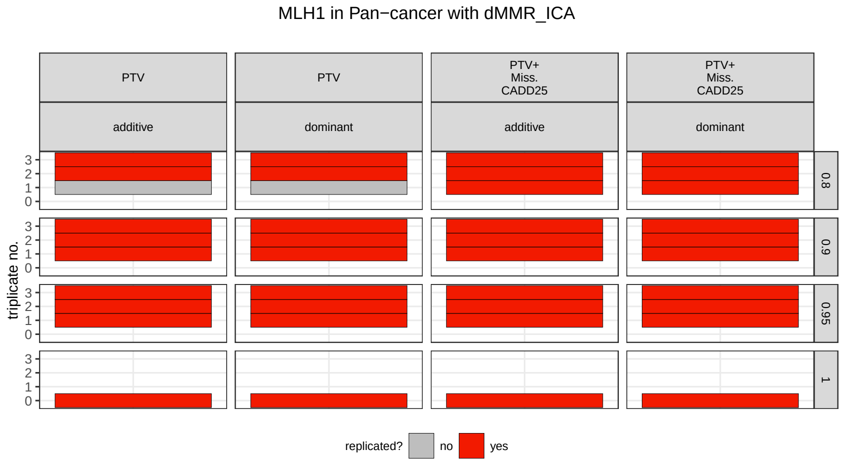

Supplementary Fig. 10: Number of replicated associations between *MLH1* and dMMR_ICA in pan-cancer in subsampling analysis. Showing replicated hits (red tile) for each pLoF rare variant set and model of inheritance combination (columns) for each subsampling (rows). Data of the validation cohort was randomly subsampled retaining 95, 90, and 80 % of the data three times for each subsampling (triplicate number on y-axis). Bottom row showing associations without any subsampling (100 % of the data).

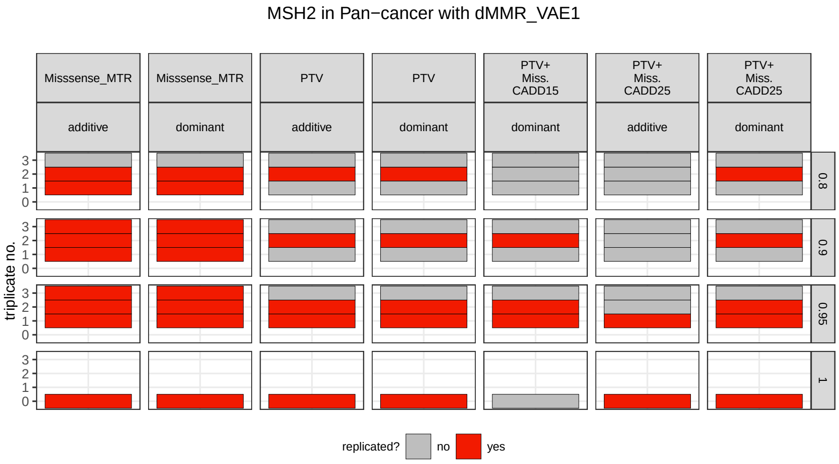

Supplementary Fig. 11: Number of replicated associations between *MSH2* and dMMR_VAE1 in pan-cancer in subsampling analysis. Showing replicated hits (red tile) for each pLoF rare variant set and model of inheritance combination (columns) for each subsampling (rows). Data of the validation cohort was randomly subsampled retaining 95, 90, and 80 % of the data three times for each subsampling (triplicate number on y-axis). Bottom row showing associations without any subsampling (100 % of the data).

2.5 Overview of Discovered Hits and/or Replicated Hits

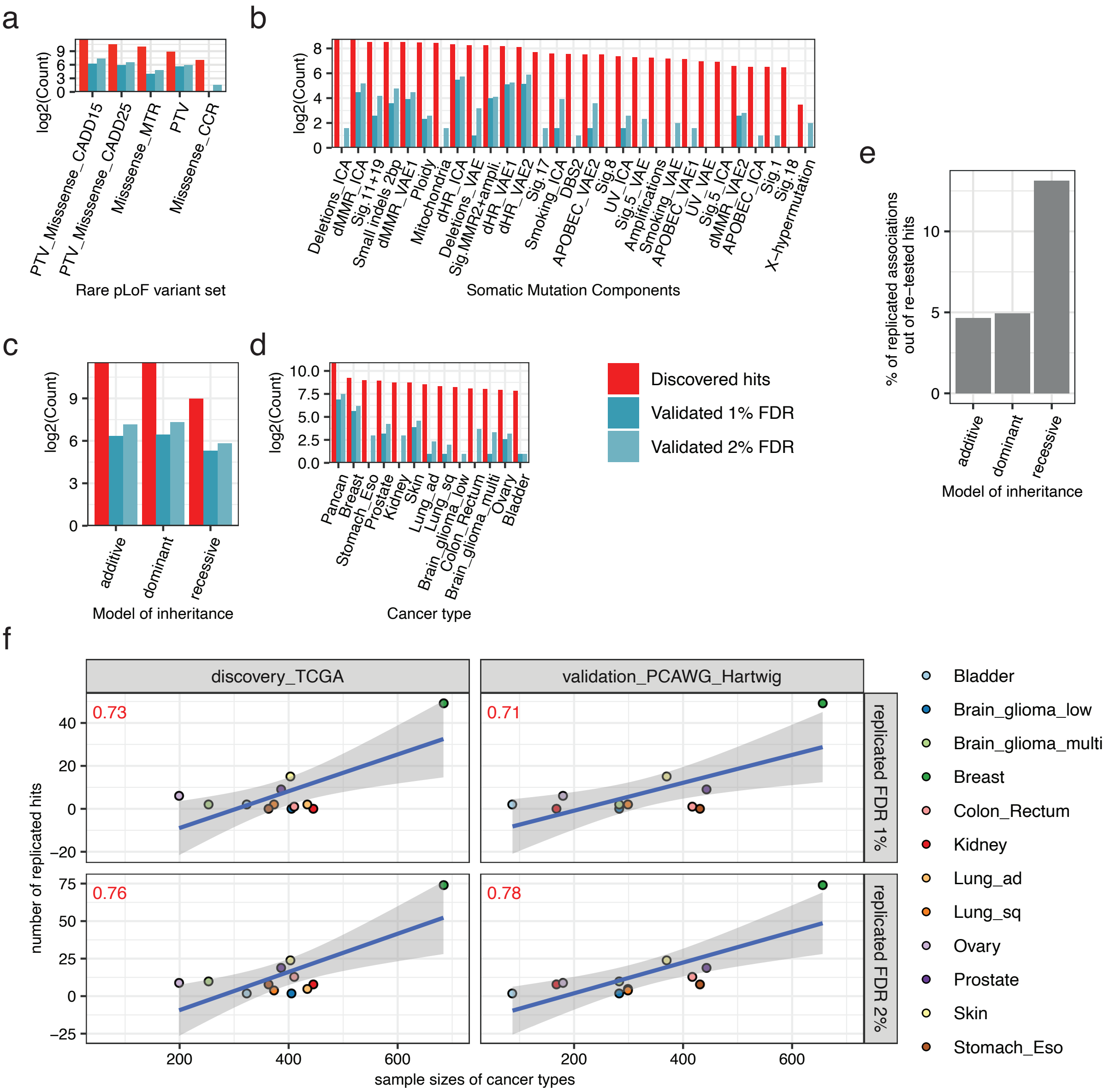

Supplementary Fig. 12: Overview of number of discovered and validated hits. a, Number of discovered hits, number of replicated hits at a FDR of 1 % and number replicated hits at a FDR of 2 % across rare pLoF variant sets, **b**, somatic components, **c**, models of inheritance, and **d**, cancer types. Log2 counts shown on the y-axis for panels a-d. **e**, Amount of replicated hits out the re-tested discovered hits at a FDR of 1 % across different models of inheritance. **f**, Number of replicated hits (y-axis) versus sample sizes of the corresponding cancer types in which they replicated (x-axis). Columns represent the two cohorts, and rows the applied FDR. Color code for the different cancer types. Pearson correlation shown on the top left corner in red and linear regression fitted through each plot (blue line). Shaded band illustrating 95 % confidence interval. Pancan analysis was excluded.

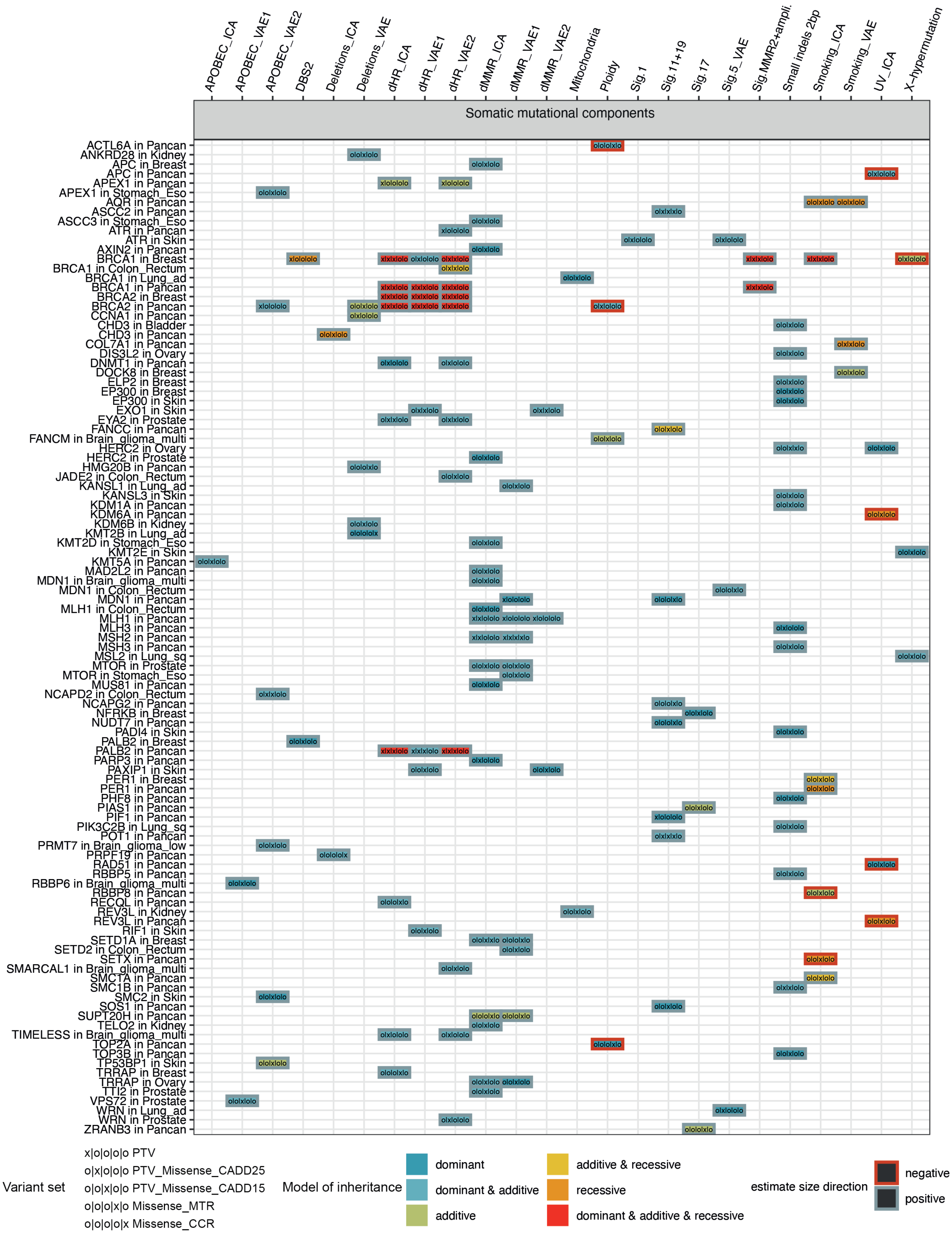

Supplementary Fig. 13: Validated hits at a FDR of 2 %. Showing replicated hits (y-axis) and the phenotype(s) they associated with (x-axis). Color code in tiles showing with which model(s) of inheritance hit associated with and symbols showing with which variant set(s) hit associated with.

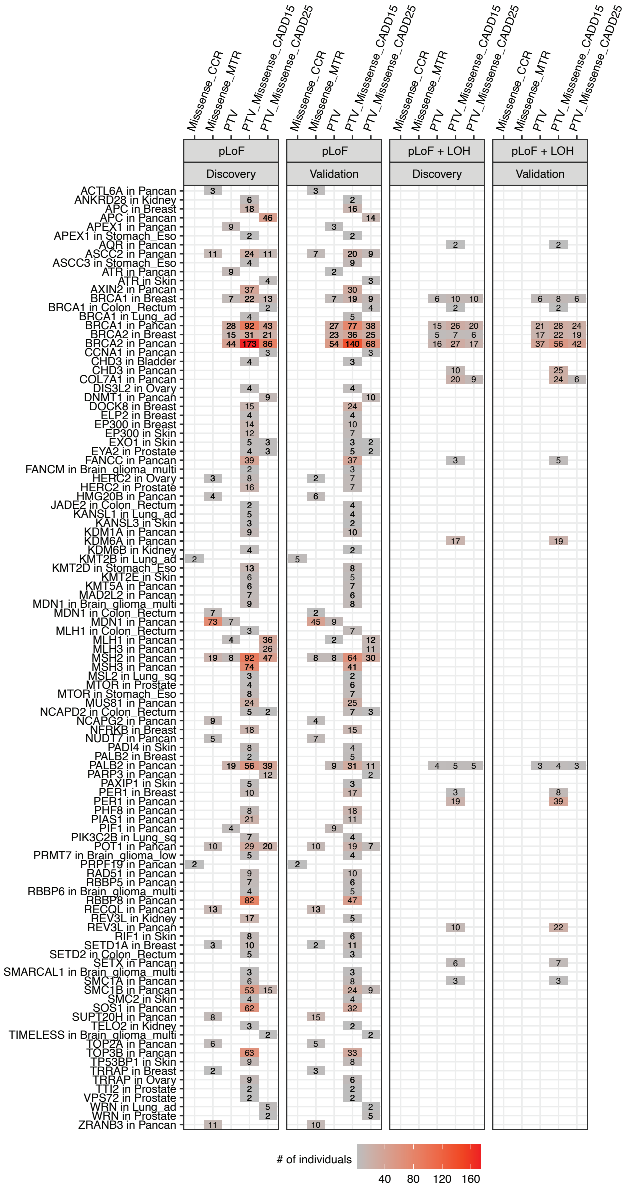

Supplementary Fig. 14: Number of individuals with rare pLOF variants (and somatic LOH) in genes replicating at a FDR of 2 %. Showing replicated hits (y-axis) and the number of individuals in the discovery (1st and 3rd column) and validation cohort (2nd and 4th column) in which these genes had rare pLOF variants (1st and 2nd column) and rare pLOF variants + somatic LOH (3rd and 4th column) for each rare pLOF variant set (x-axis). Number of individuals written in each tile for each combination for which gene associated with.

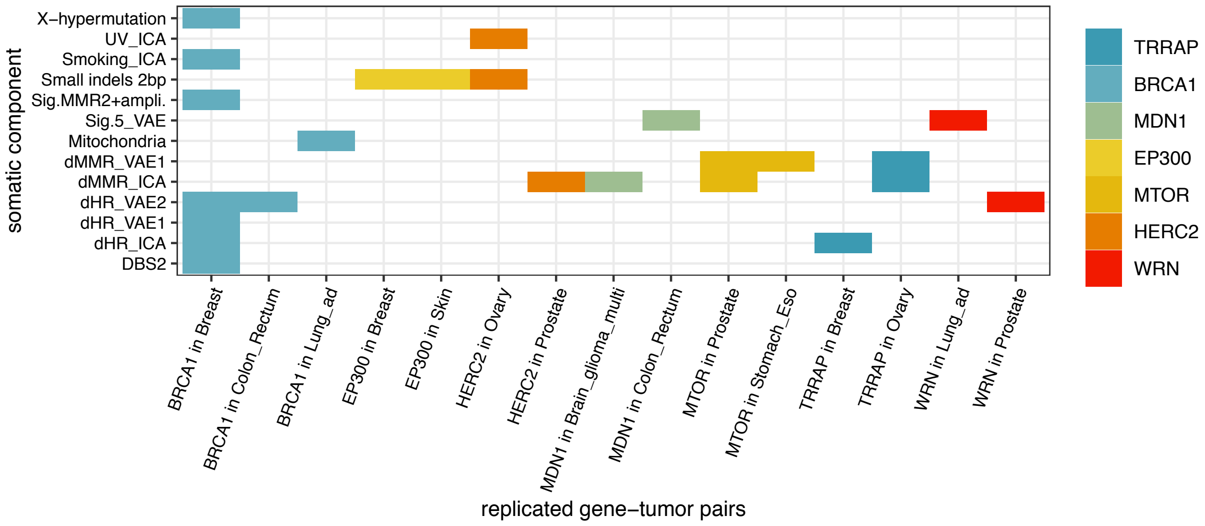

Supplementary Fig. 15: Seven genes associated with a somatic component in $\geq$ 1 cancer type. Showing gene-cancer type pairs (x-axis) and the corresponding somatic component they associated with at a FDR of 2 % (y-axis). Color code for each gene. Results from pancan analysis excluded.

2.6 Distributions of Rare Variants

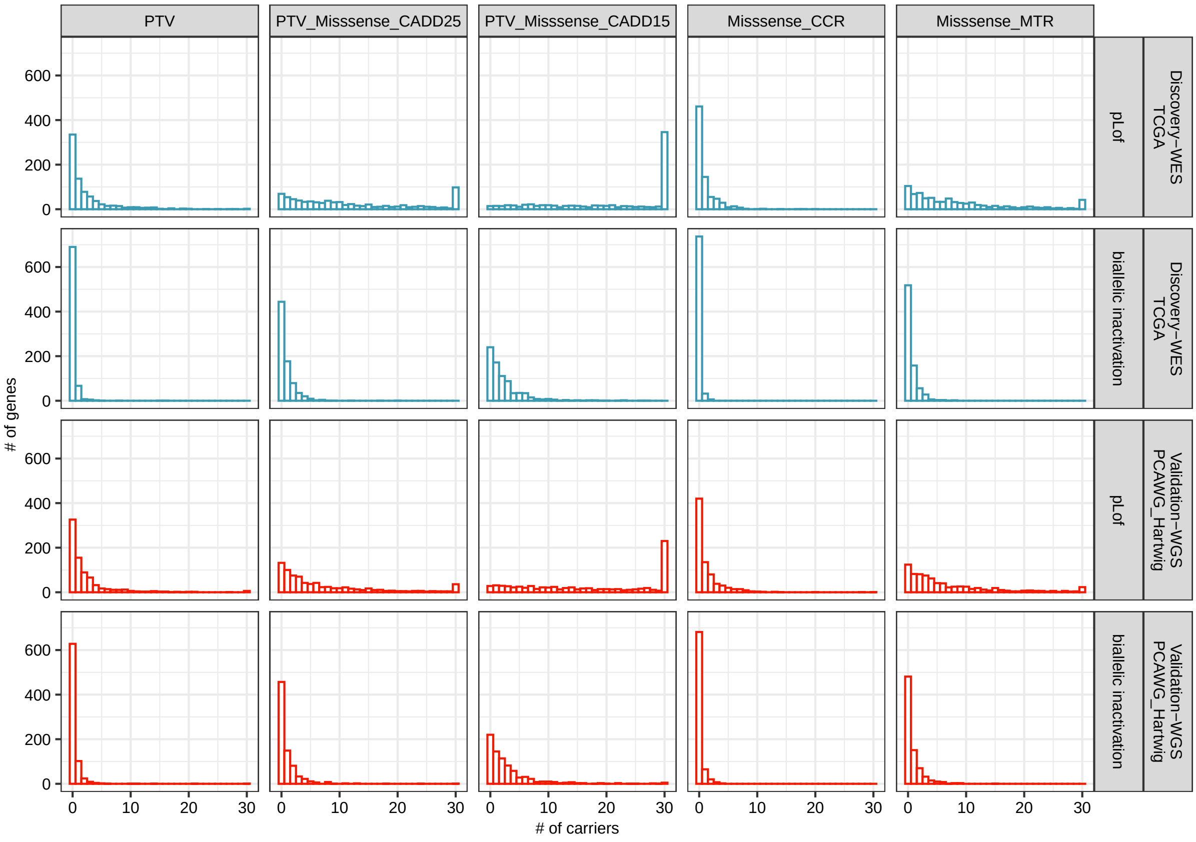

Supplementary Fig. 16: Overall occurrence of pLOF variants and pLOF variants + somatic LOH in the cohorts. Showing in how many individuals (x-axis) how many genes (y-axis) were identified with pLOF variants (1st and 3rd row) and pLOF variants + somatic LOH (2nd and 3rd row). Distributions are shown separately for the discovery (1st and 2nd row) and validation (3rd and 4th row) cohort and distributions are shown for each definition of pLOF (columns). Values were capped at 30 for this visualisation. pLOF variants of somatic LOH in the same gene were considered as biallelic inactivations.

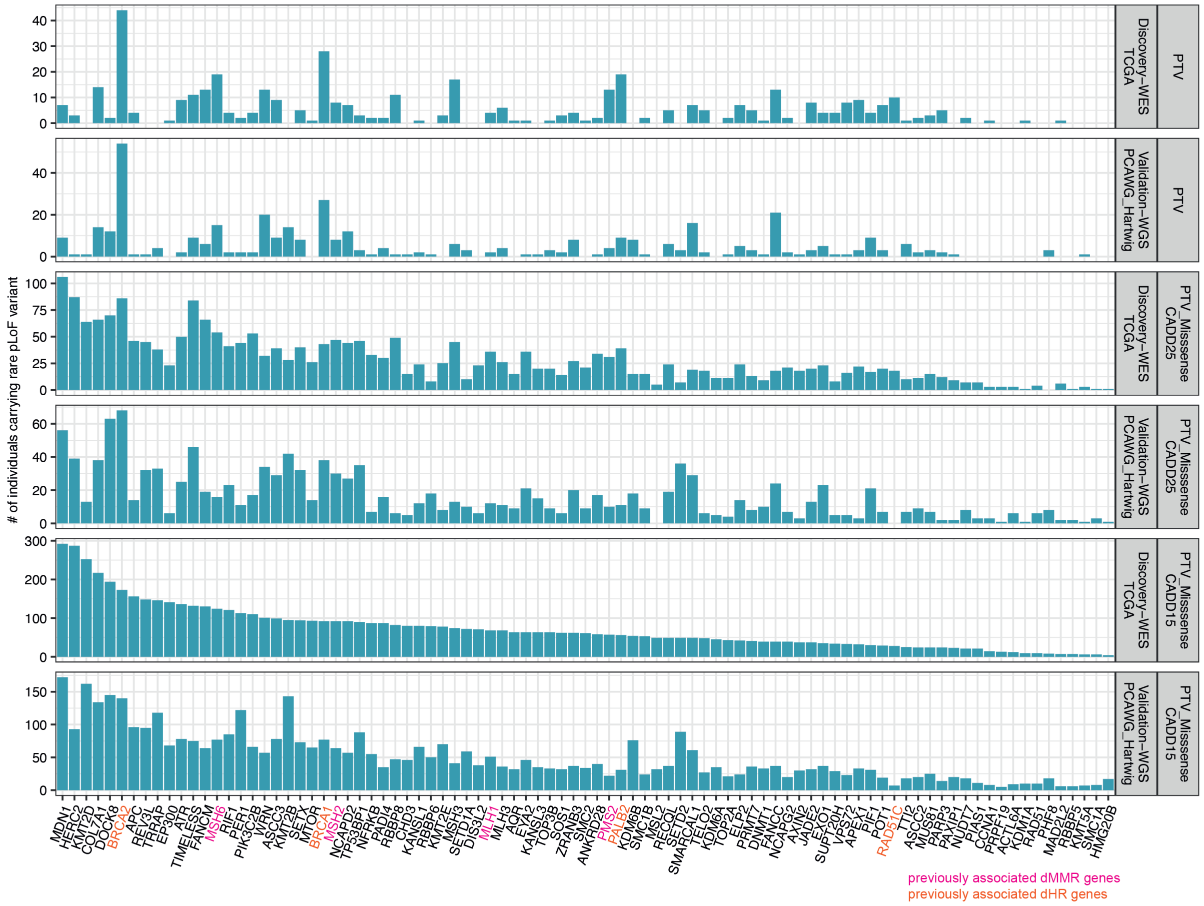

Supplementary Fig. 17: Number of individuals carrying a rare pLOF variant in one of the genes replicating at a FDR of 2 %. Showing the number of individuals (y-axis) carrying a rare pLOF variant in one of the 86 replicated genes or *PMS2*, *MSH6*, and *RAD51C* (x-axis). Showing numbers separately for the discovery and validation cohort in combination with three utilized definitions of pLOF.

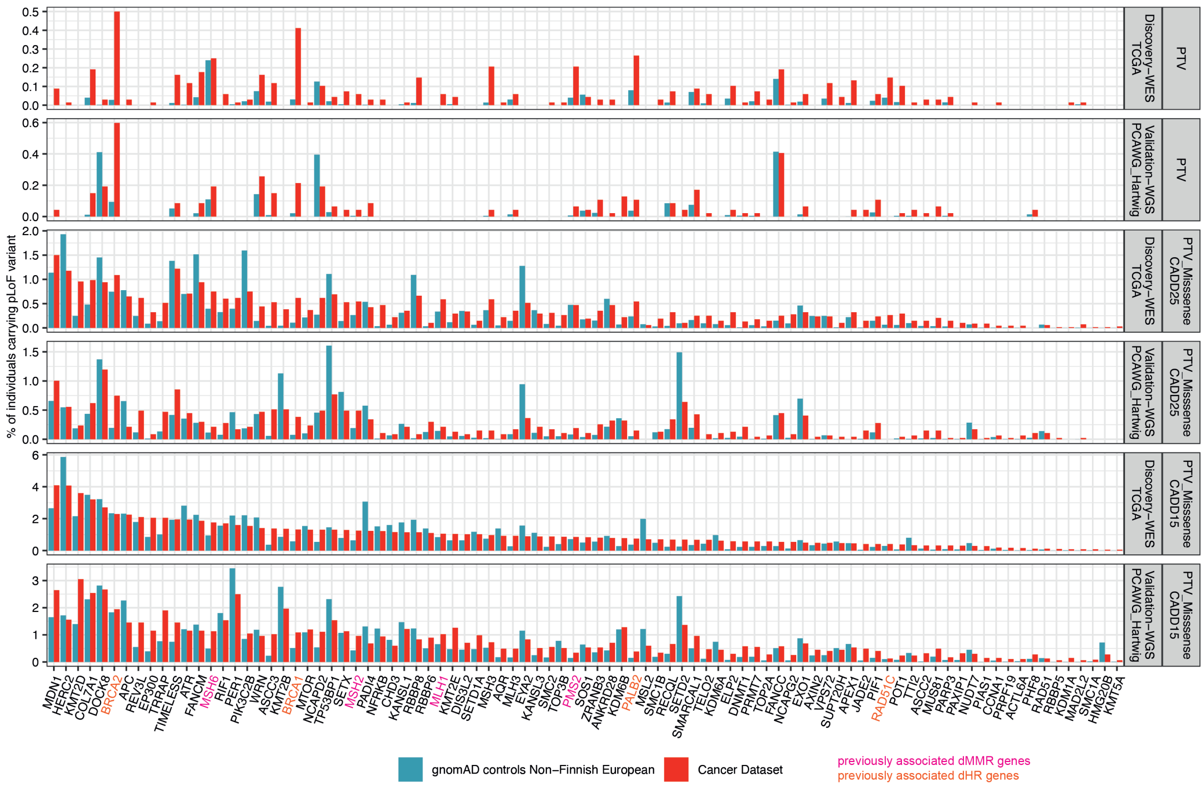

Supplementary Fig. 18: Frequency of pLOF variants in analyzed cancer dataset vs. control samples in gnomAD v2.1[12]. Showing frequency of rare pLOF variants (y-axis) based on three different definitions and for the discovery and validation cohort separately (rows) as red bars for all 86 genes which replicated at a FDR of 2 % in addition to *PMS2*, *MSH6*, and *RAD51C*. In comparison, showing frequency of the same variants identified in the cancer datasets (red bard) in all gnomAD control samples with Non-Finnish European ancestry (blue bar). Control samples in gnomAD comprise samples which did not have a common disease and/or were included as control samples in studies. For this analysis, in the cancer datasets only rare pLOF variants were included which were also identified in gnomAD v2.1.

2.7 Network Analysis

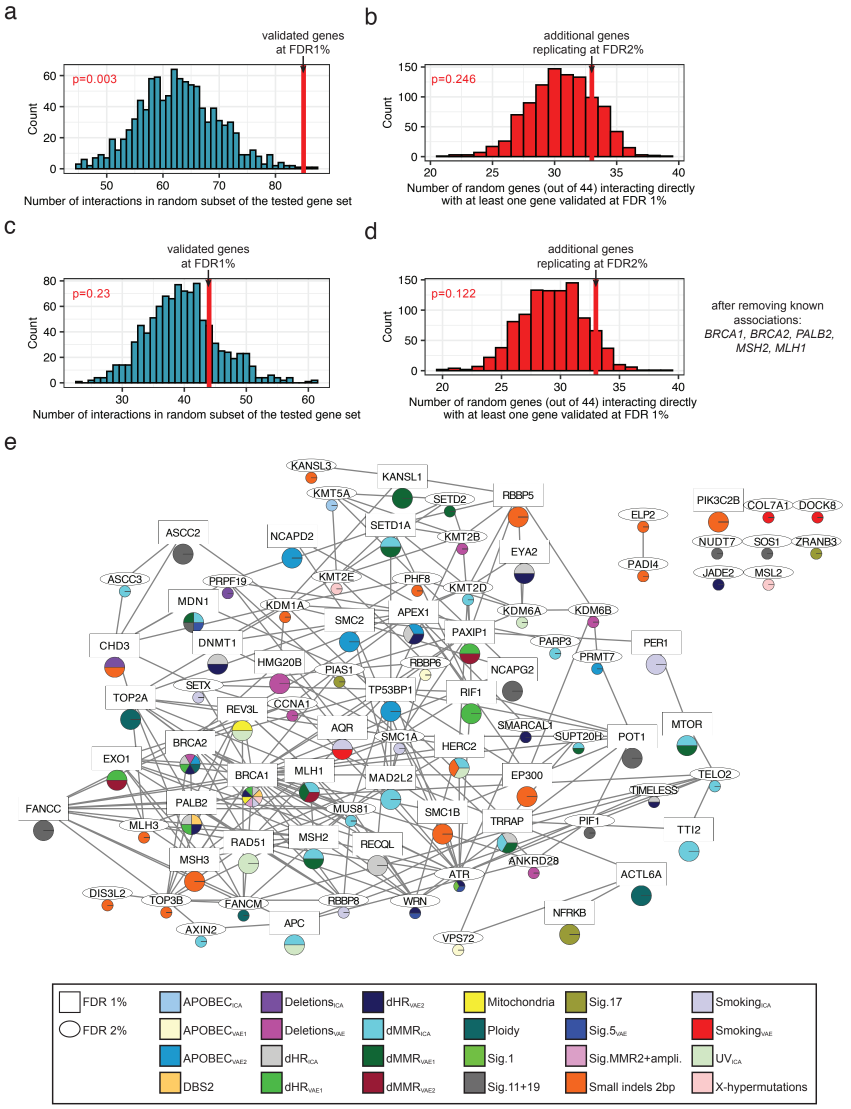

Supplementary Fig. 19: Network analysis (HumanNet) supports the role of rare germline variation in somatic mutational processes. All panels in this figure were generated using the functional gene network from HumanNet. In panels a-d p-values were calculated via randomization using a one-sided test. **a**, Number of physical interactions in a random subset of the tested gene set (controlled for interaction node degree) (x-axis) (blue bars). Red line shows the number of interactions within genes which replicated at a FDR of 1 %. **b**, Number of randomly selected genes from the tested gene set interacting with at least one gene, which replicated at a FDR of 1 % (x-axis) (red bars), (controlled for interaction node degree). Randomization performed 1,000 times. Red line shows the number of genes, out of the ones which additionally replicated at a FDR of 2 %, interacting with at least one gene replicating at a FDR of 1 %. **c**, Same as in a after excluding known genes from the analysis (*BRCA1*, *BRCA2*, *PALB2*, *MSH2*, and *MLH1*). **d**, Same as in b after excluding known genes from the analysis (*BRCA1*, *BRCA2*, *PALB2*, *MSH2*, and *MLH1*). **e**, Visualisation of interactions between proteins for genes replicating at a FDR of 1 % (square) and genes replicating at a FDR of 2 % (ellipse). Color code in pie chart showing the somatic components the corresponding gene associated with. Line width corresponding to interaction score.

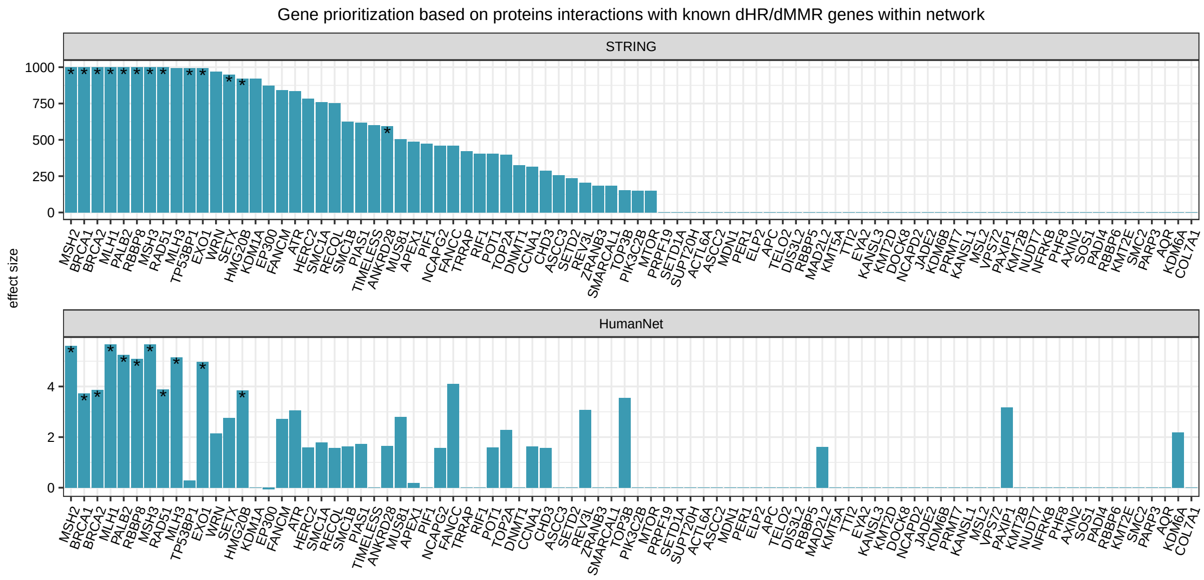

Supplementary Fig. 20: Gene prioritization based on protein interactions with known dHR/dMMR genes within network. Within a network it was estimated how strongly a protein interacts with a known dHR (BRCA1, BRCA2, PALB2, RAD51C) or MMR (MLH1, MSH2, MSH6, PMS2) protein in comparison to other proteins with the same node connectivity. Gene prioritization was performed with STRING network (top) and HumanNet (bottom). Genes reaching significance at a FDR of 25 % were marked with an asterisk. Mulitple testing correction was performed via Benjamini-Hochberg procedure.

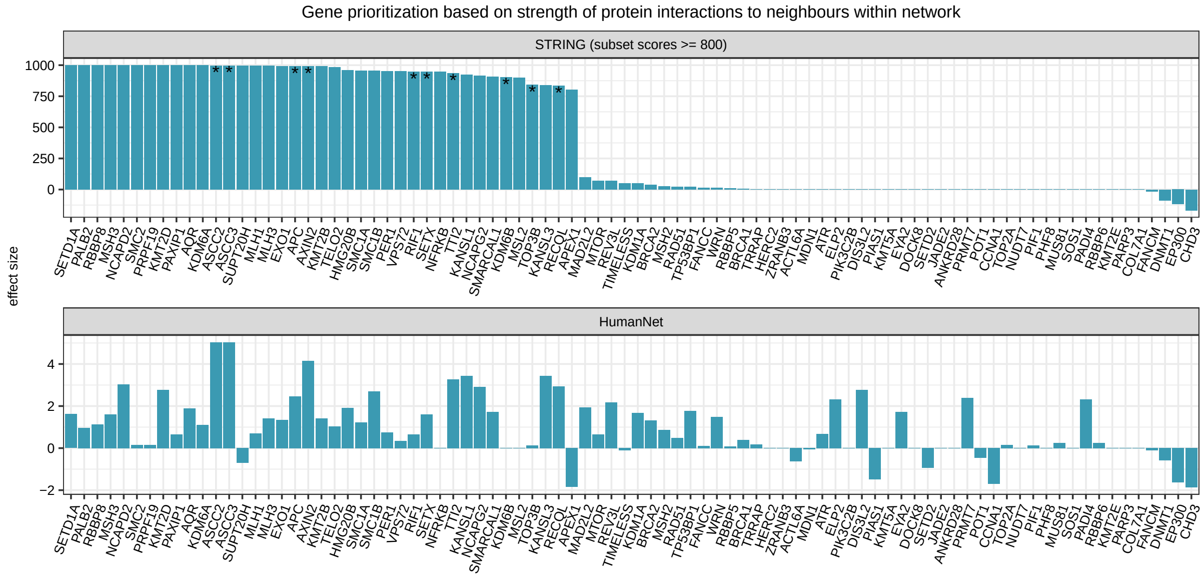

Supplementary Fig. 21: Gene prioritization based on strength of protein interactions to neighbours within network. Within a network it was estimated whether a protein had stronger interactions with its neighbours than with its neighbours within a random network with the same node connectivity. Gene prioritization was performed with STRING (subset with interactions having a score $\geq$ 800) network (top) and HumanNet (bottom). Genes reaching significance at a FDR of 25 % were marked with an asterisk. Mulitple testing correction was performed via Benjamini-Hochberg procedure.

2.8 Somatic Input Features

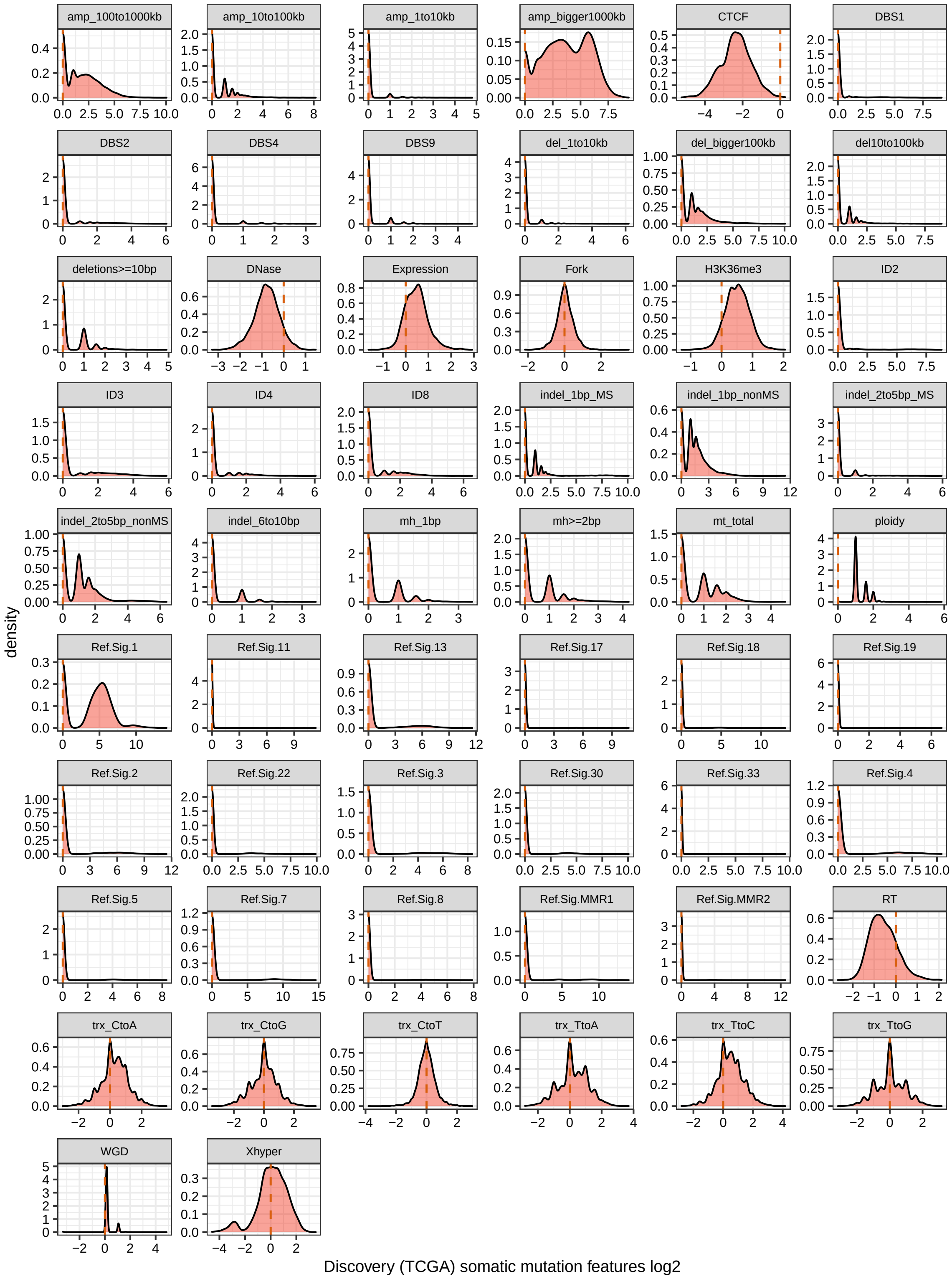

Supplementary Fig. 22: Distribution of all 56 somatic features in TCGA-WES. Dashed red line going through 0. Fork: replicative strand bias, RT: replication timing, trx: transcription strand bias, Xhyper: Chromosome X hypermutation.

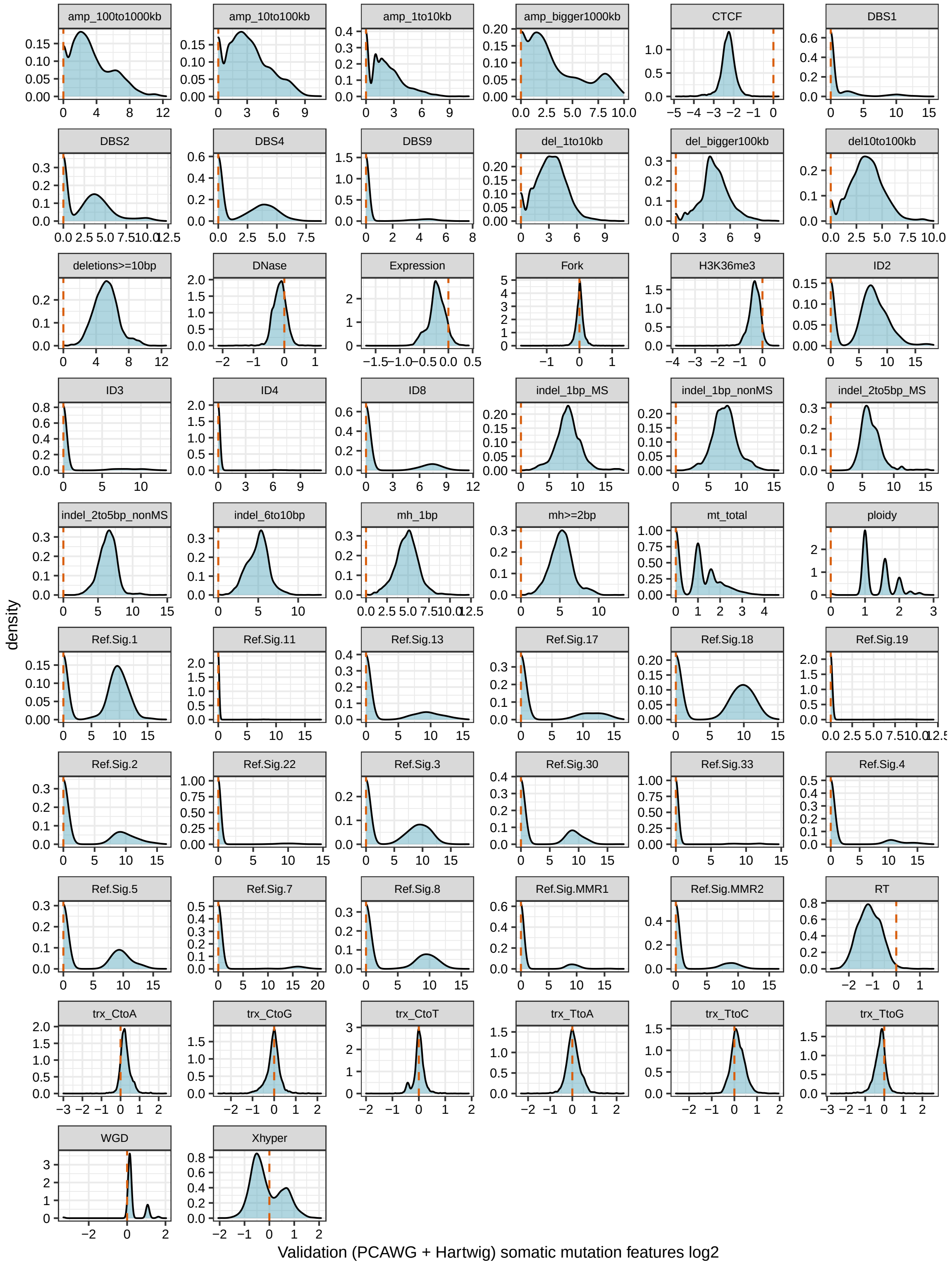

Supplementary Fig. 23: Distribution of all 56 somatic features in PCAWG_Hartwig -WGS. Dashed red line going through 0. Fork: replicative strand bias, RT: replication timing, trx: transcription strand bias, Xhyper: Chromosome X hypermutation.

2.9 Extraction of Independent Components

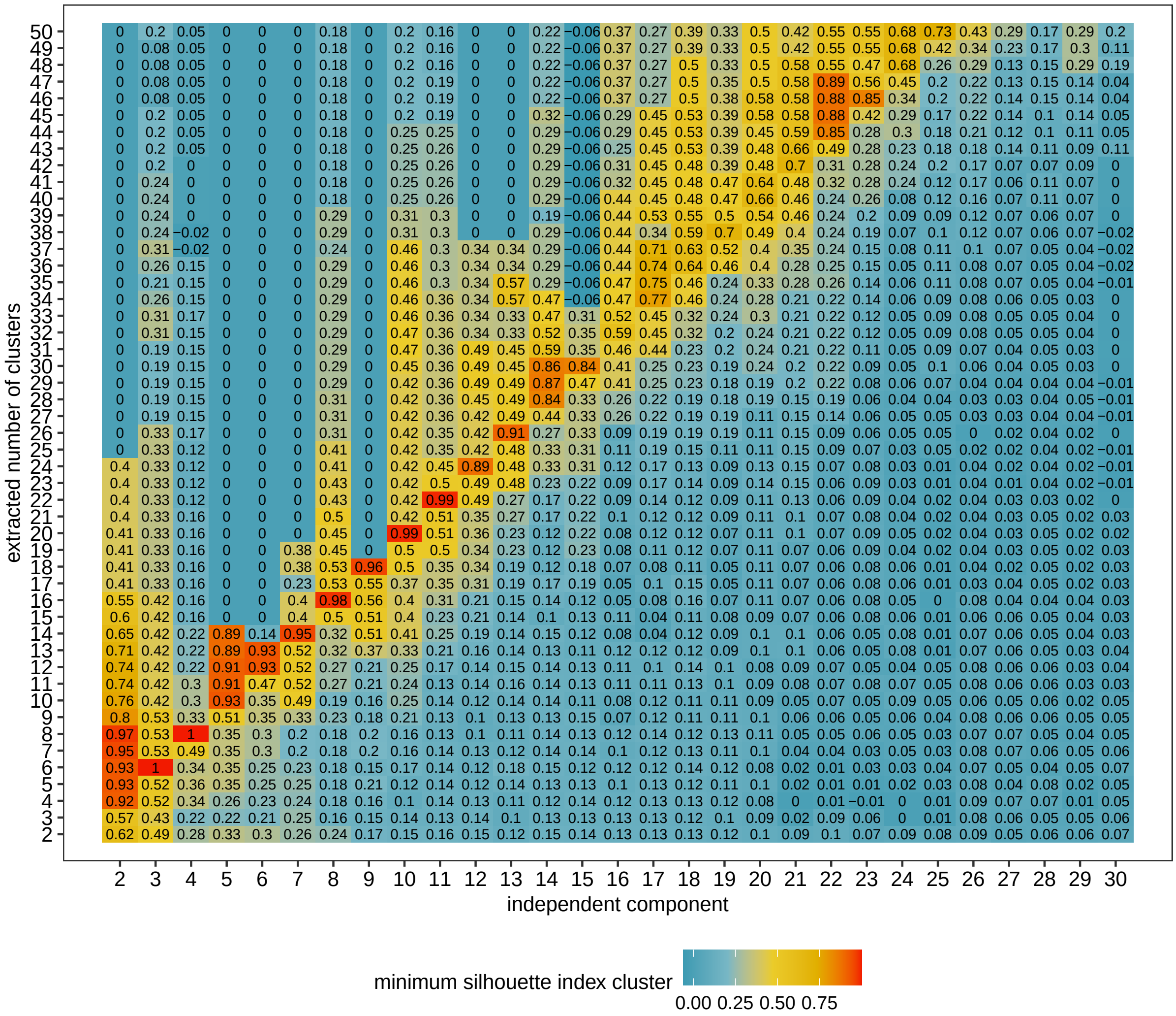

Supplementary Fig. 24: Finding the optimal number of independent components. Independent component extraction was run with increasing number of independent components (x-axis). Each extraction was performed 200 times and then clustered using k-medoid clustering with increasing number of clusters (y-axis). Color code and number in each tile shows the minimum silhouette index of a cluster.

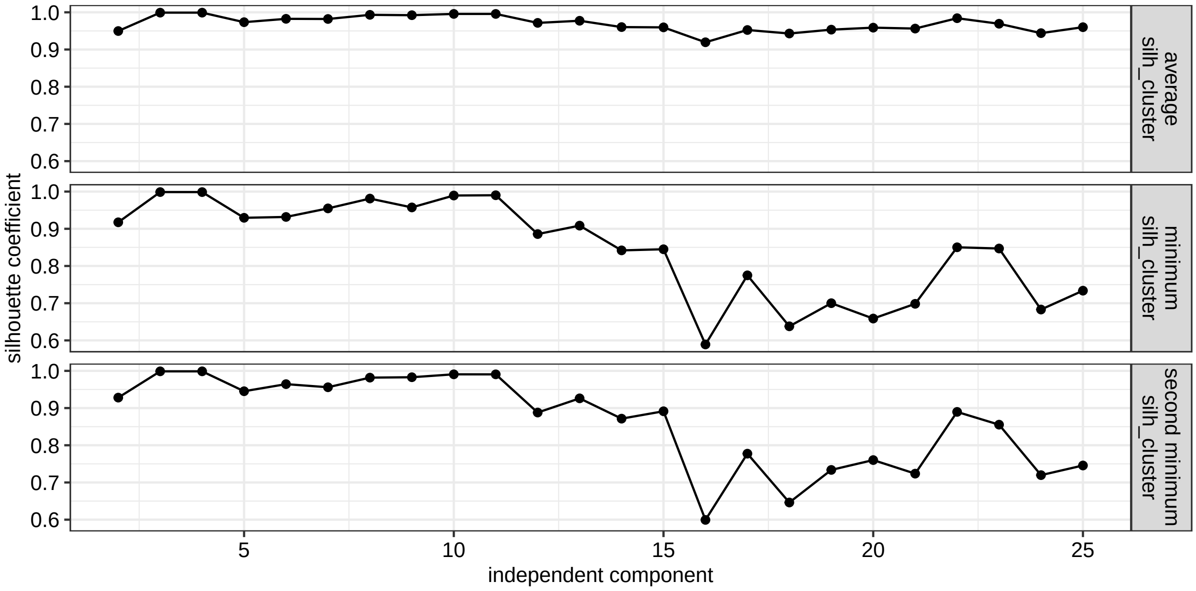

Supplementary Fig. 25: Selection of 15 independent components for further analysis. Showing the average, minimum and second minimum silhouette index of the clusters when extracting 2 times more clusters for a set number of components.

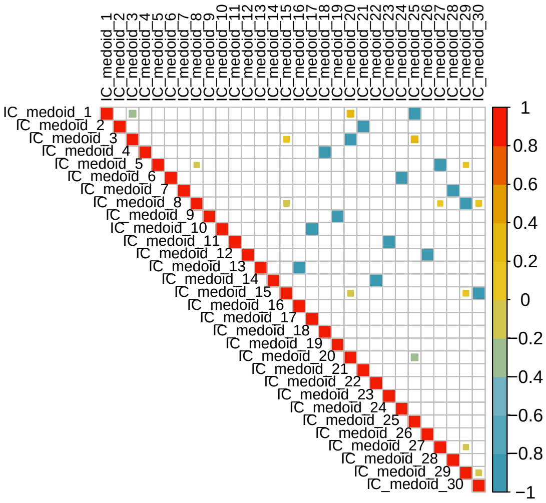

Supplementary Fig. 26: Pearson correlations between all 30 independent components which were extracted using 15 components and k-medoid clustering with k = 30. Each component occurred twice with opposite signs.

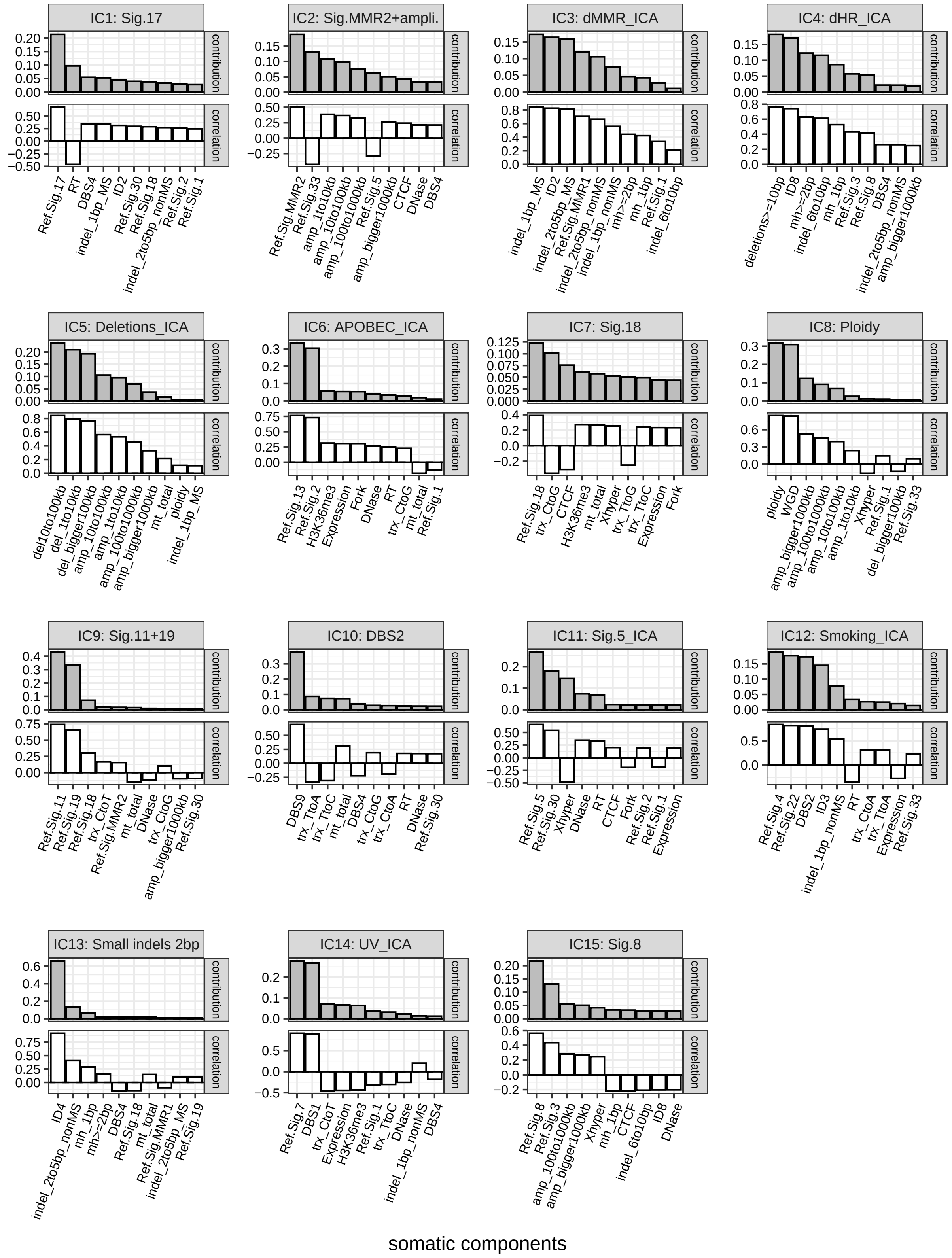

Supplementary Fig. 27: Overview of strongest contributing features to the independent components. Showing the Pearson correlation (white bars) and contribution (fraction of 1) (grey bars) of the 10 strongest somatic features to the respective components. Fork: replicative strand bias, RT: replication timing, trx: transcription strand bias, Xhyper: Chromosome X hypermutation. Components were renamed based on strongest correlating somatic features.

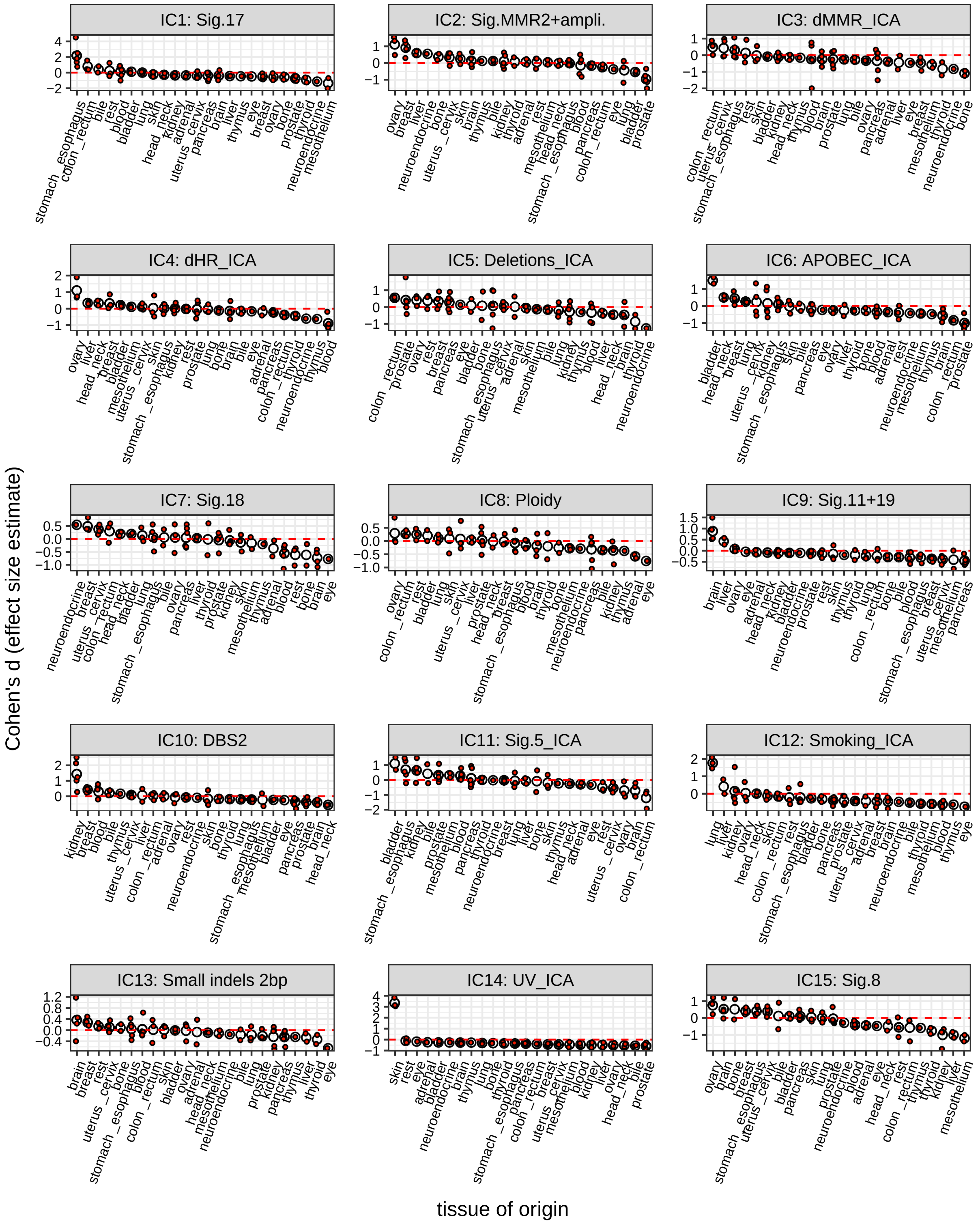

Supplementary Fig. 28: Several independent component scores were enriched in specific tissue of origins. Cohen's *d* (effect size estimate) was calculated for each cancer type, grouped by tissue of origin and, then the average value was estimated. Average effect size estimates were ordered by decreasing value for each independent component. Components were renamed based on strongest correlating somatic features. Dashed red line going through 0.

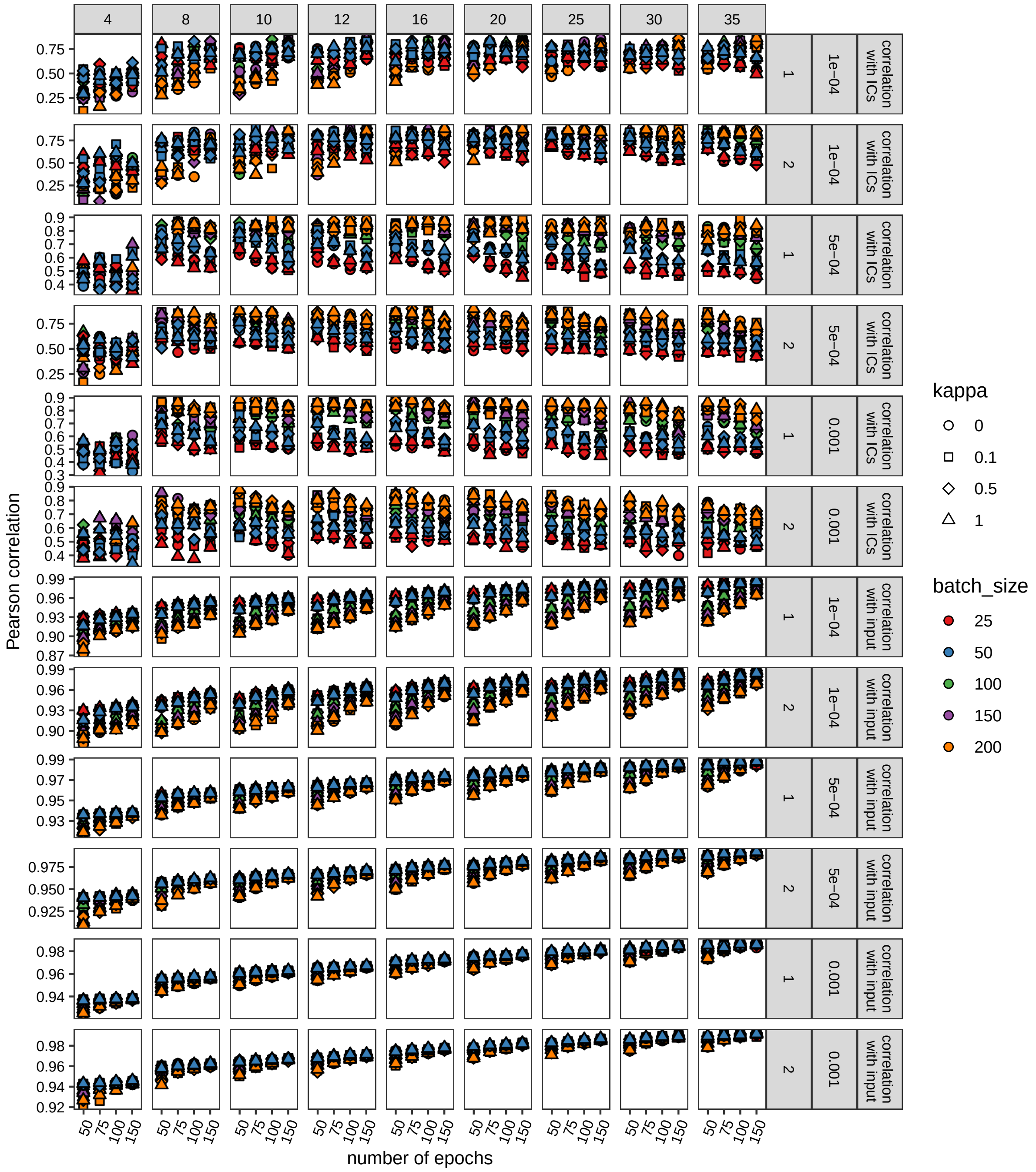

Supplementary Fig. 29: Finding the optimal hyperparameters for the variational autoencoder. Increasing number of epochs (x-axis), different kappa factors (point shape), increasing batch sizes (point color), three different learning rates (0.001, 0.0005, and 0.0001), different number of hidden layers between latent space and input/output (either 1 or 2) and different number of components (4, 8, 10, 12, 16, 20, 25, 30, and 35) were tested. Evalutation by measuring the average Pearson correlation with 4 ICs (UV, smoking, dMMR, and dHR ICs) and by calculating the Pearson of the reconstructed input with the initial input (y-axis).

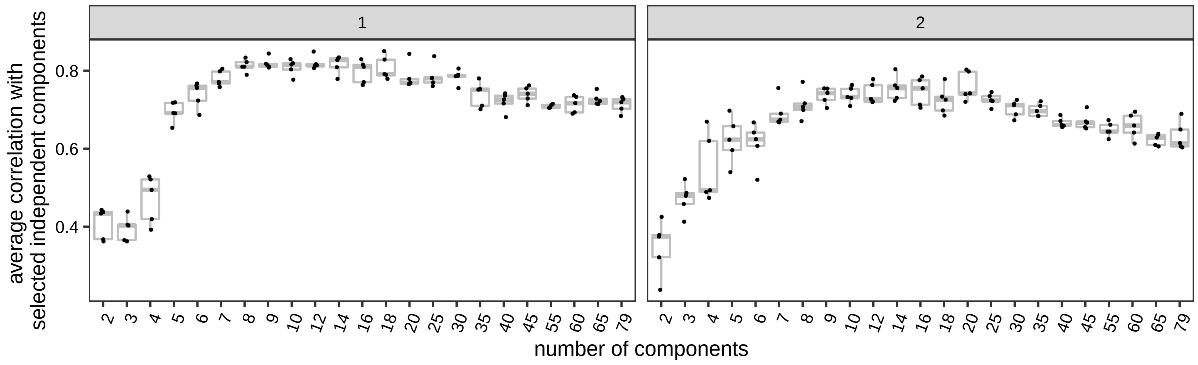

Supplementary Fig. 30: Correlation with biologically relevant components increased with increasing number of component extractions and quickly reached saturation. Component extractions were run 5 times for each set component number with different random initiations. Number of components (x-axis) are shown against the average Pearson correlation with 4 biologically relevant IC components (UV, smoking, dMMR, dHR). Facet for either using 1 hidden layer or 2 hidden layers between latent space and input/output. Centre of each boxplot shows median value, bounds of box at 25th and 75th percentiles and minimum and maximum extend to the smallest and largest value, but not more than 1.5 times the interquartile range.

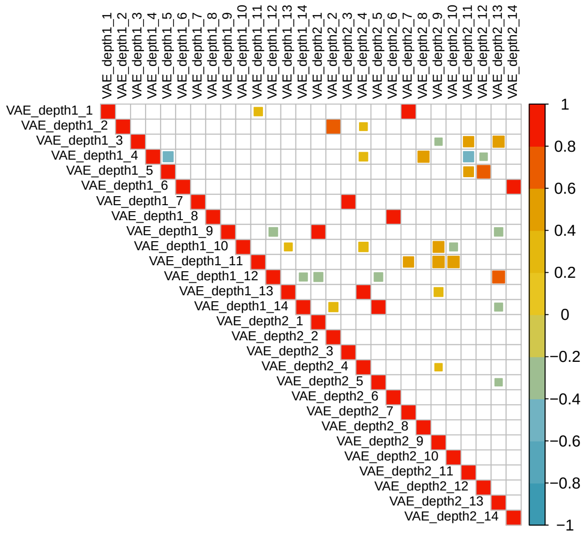

Supplementary Fig. 31: Number of hidden layers barely made a difference on the extracted components in the latent space of the variational autoencoder. Pearson correlation between the 14 extracted components with the variational autoencoder using either 1 hidden layer (depth = 1) or 2 hidden layers (depth = 2) between latent space and input/output.

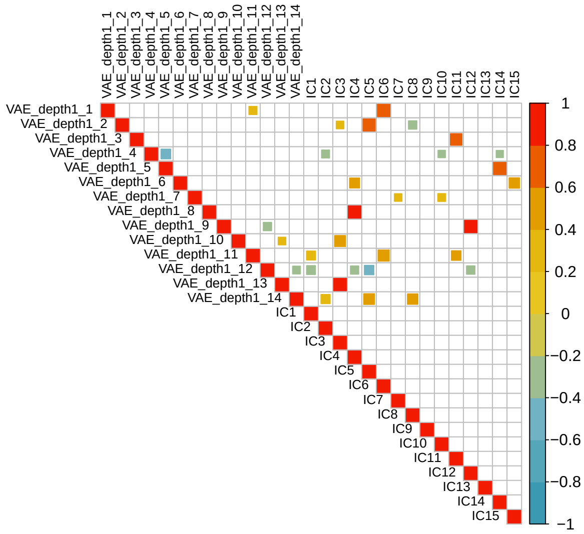

Supplementary Fig. 32: Some VAE-derived components were not captured in the independent component analysis. Showing the Pearson correlation between the VAE-derived components using 1 hidden layer (depth = 1) and the ICs.

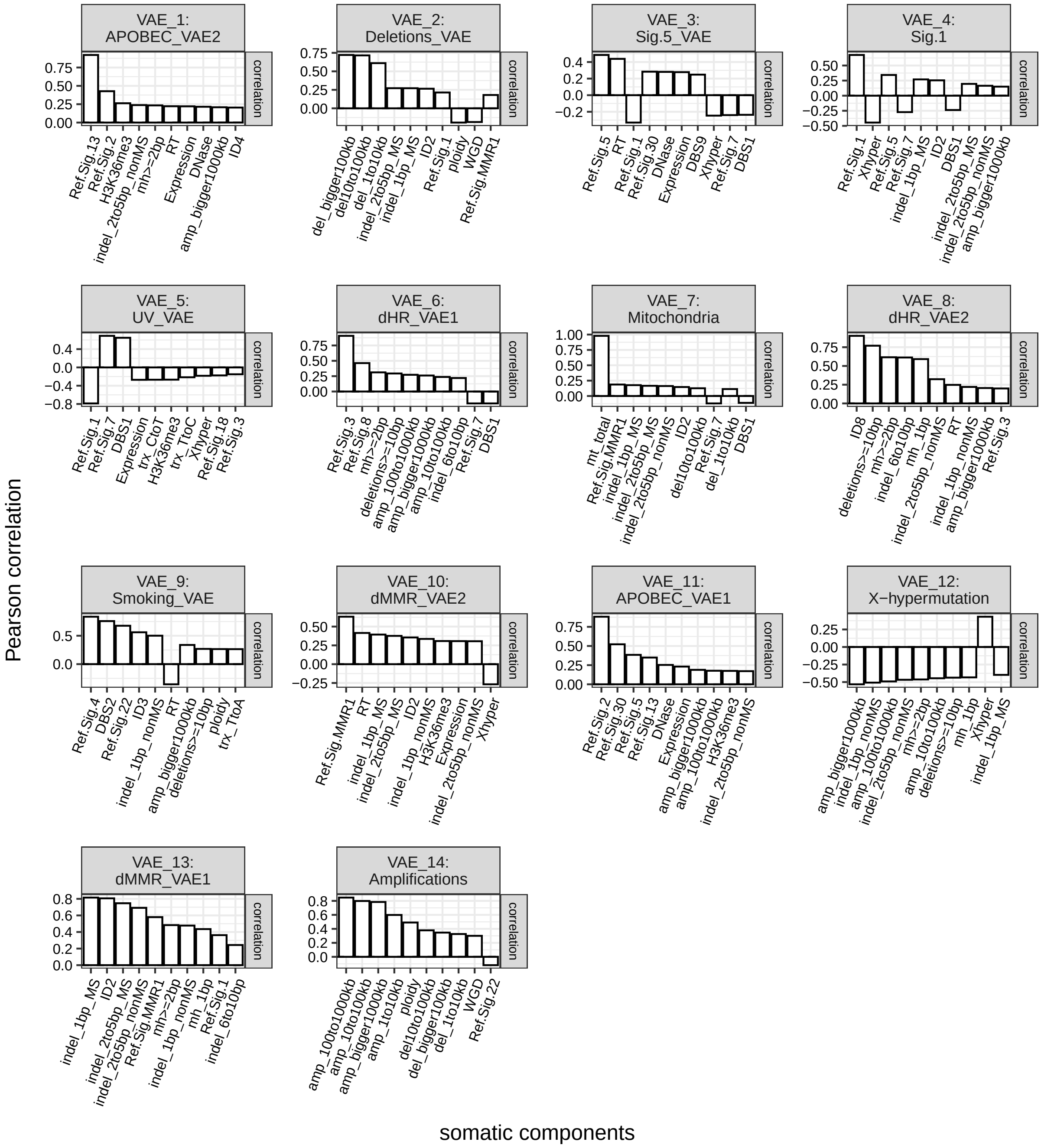

Supplementary Fig. 33: Overview of strongest contributing features to the variational autoencoder derived components. Showing the Pearson correlation (white bars) of the 10 strongest somatic features to the respective components, which were extracted via 1 hidden layer between latent space and input/output. Fork: replicative strand bias, RT: replication timing, trx: transcription strand bias, Xhyper: Chromosome X hypermutation. Components were renamed based on strongest correlating somatic features.

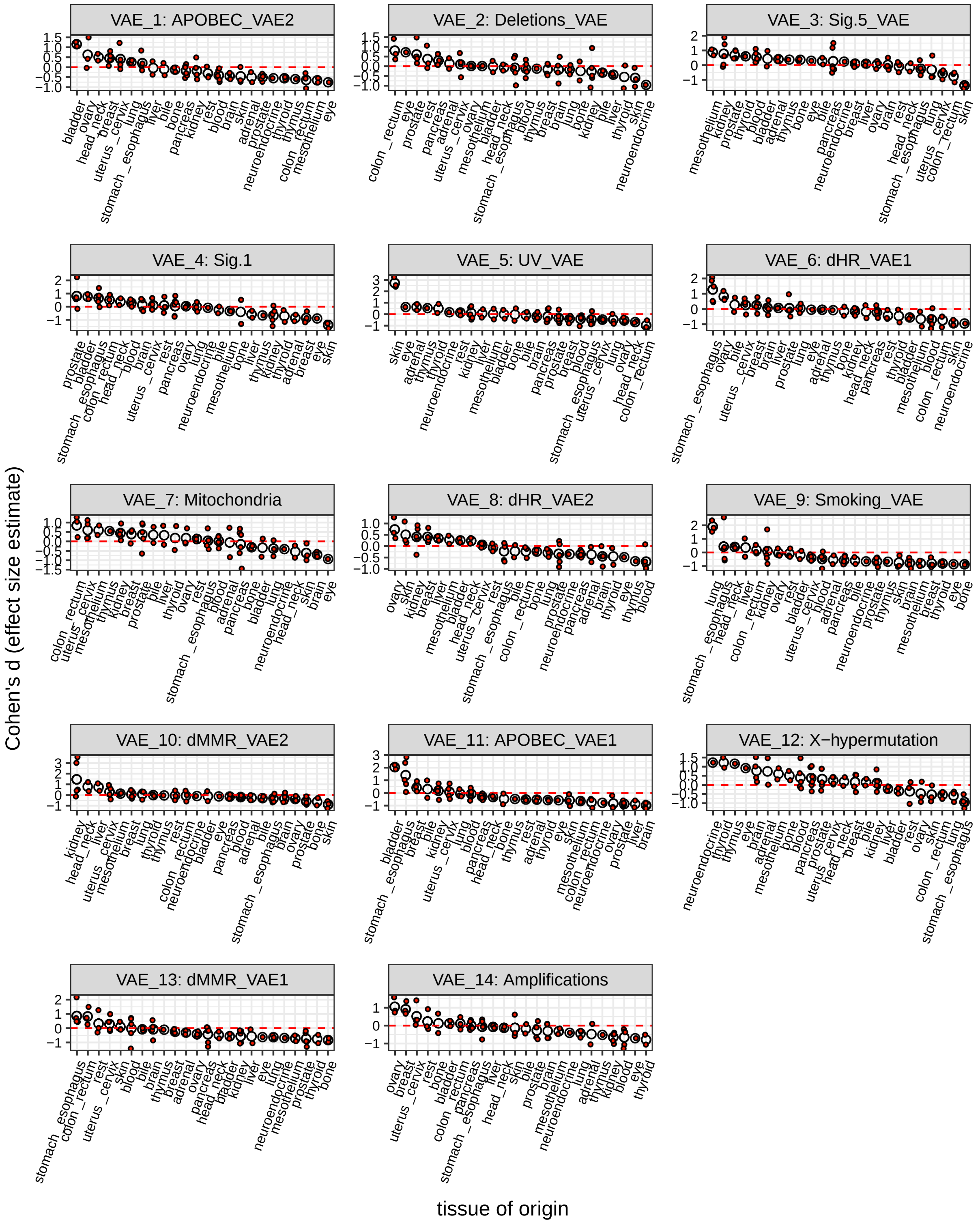

Supplementary Fig. 34: Several VAE-derived component scores were enriched in specific tissue of origins. Cohen's *d* (effect size estimate) was calculated for each cancer type, grouped by tissue of origin and, then the average value was estimated. Average effect size estimates were ordered by decreasing value for each VAE-derived component. Components were renamed based on strongest correlating somatic features. Dashed red line going through 0.

2.11 Overview of 29 Extracted Components

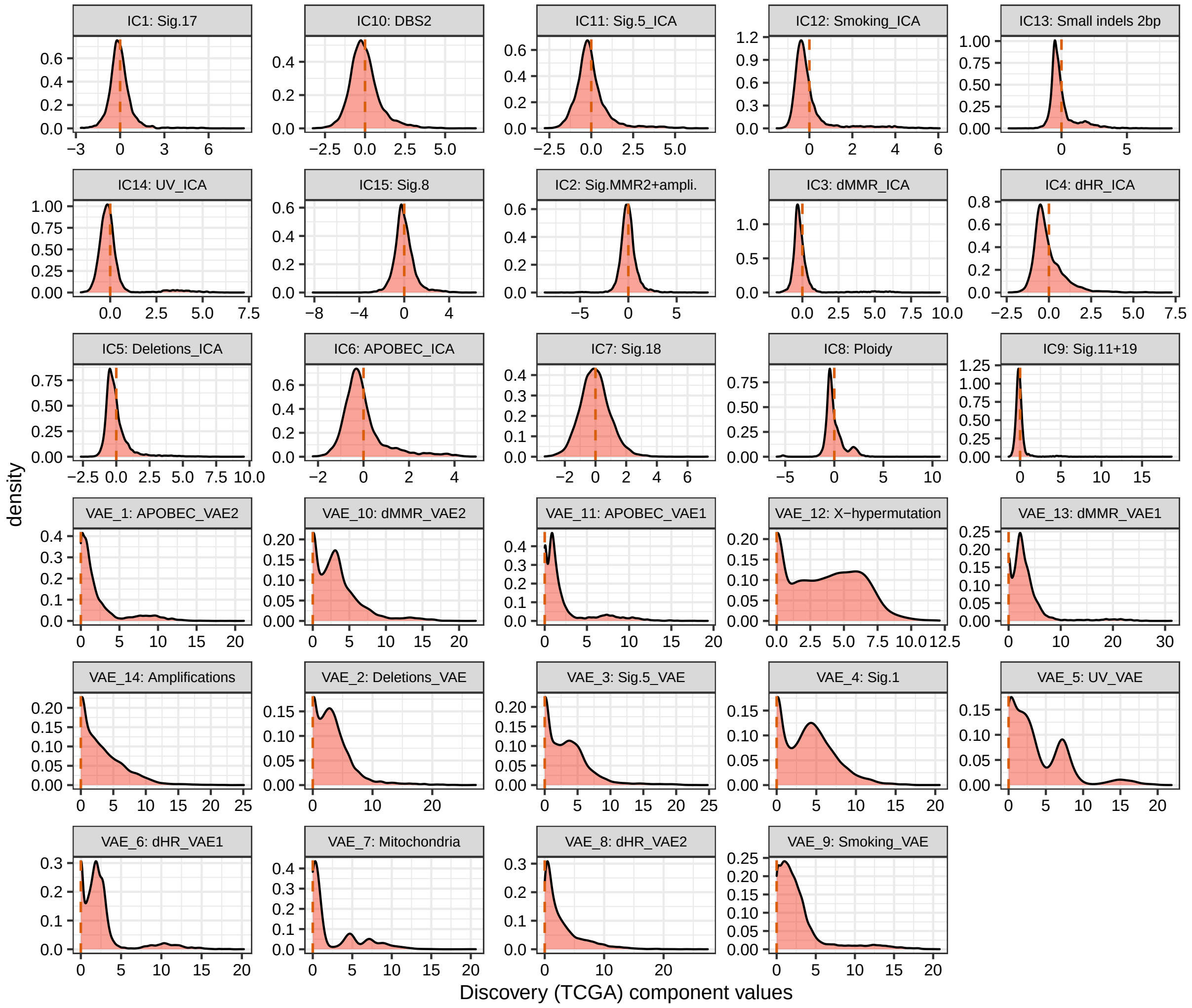

Supplementary Fig. 35: Distribution of all 29 somatic components in TCGA -WES. Showing distribution of values of all extracted ICA and VAE components in the discovery cohort TCGA -WES. Components were renamed based on strongest correlating somatic features. Dashed red line going through 0.

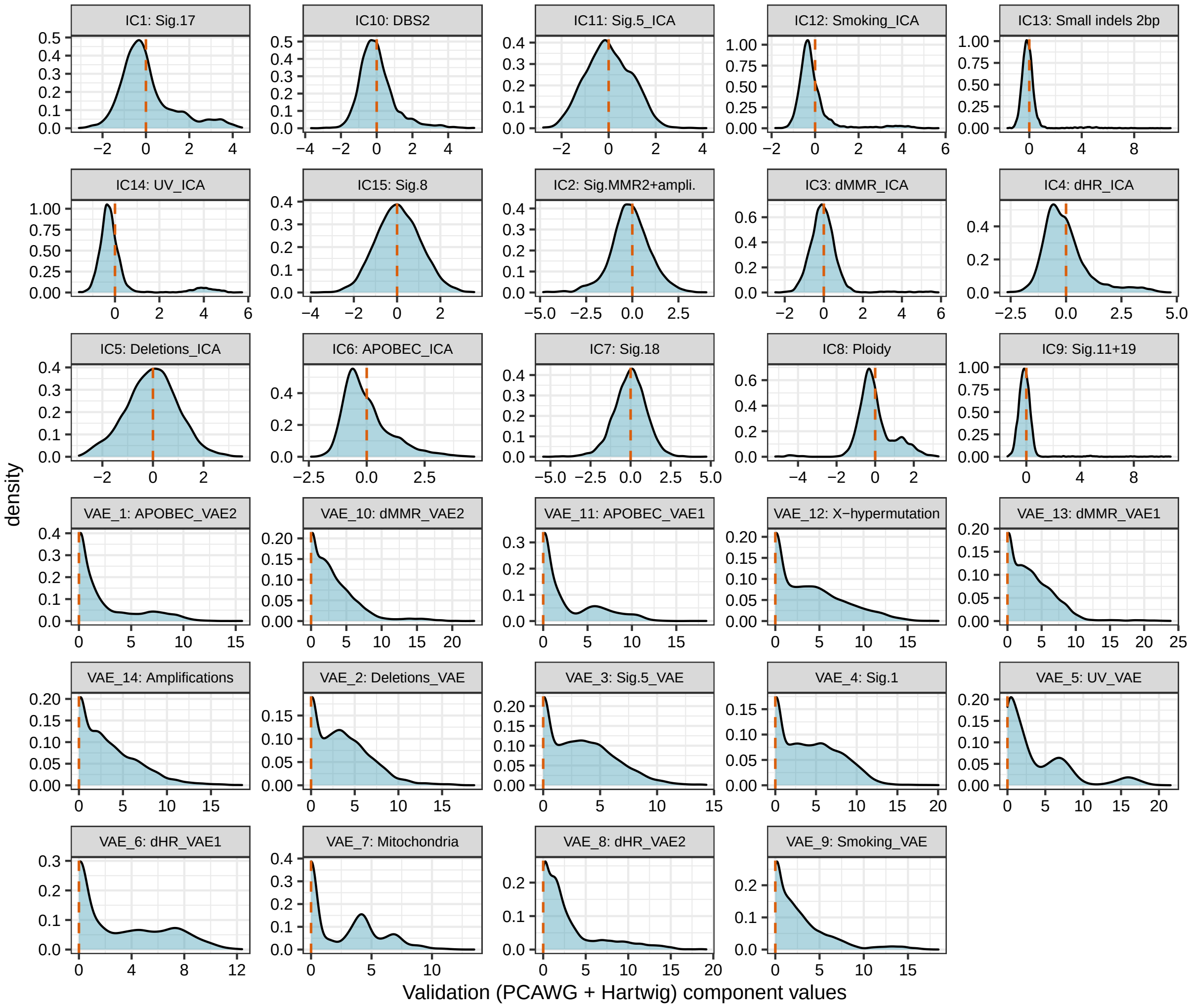

Supplementary Fig. 36: Distribution of all 29 somatic components in PCAWG_Hartwig-WGS. Showing distribution of values of all extracted ICA and VAE components in the validation cohort PCAWG_Hartwig-WGS. Components were renamed based on strongest correlating somatic features. Dashed red line going through 0.

2.12 Correlation between IC9 (Sig.11+19) and telomere features

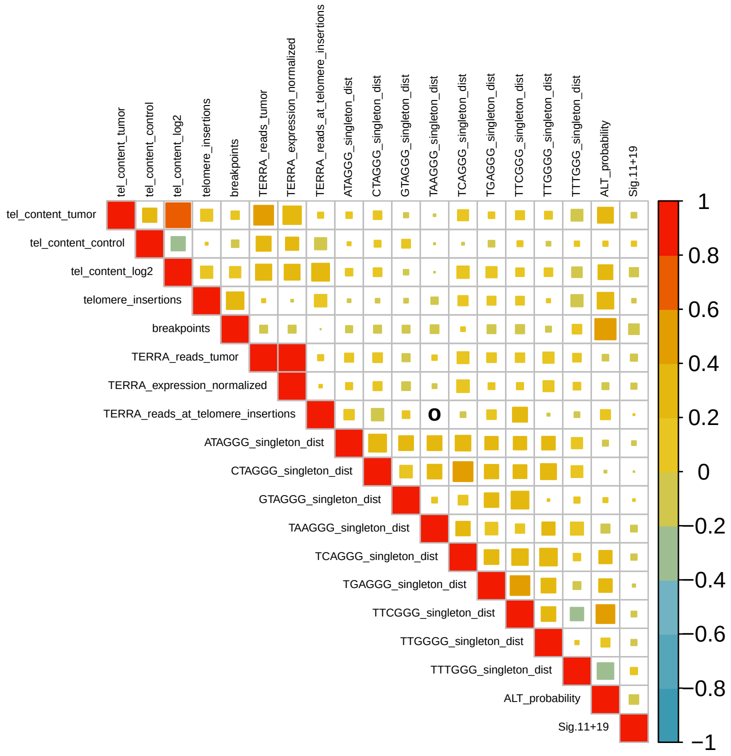

Supplementary Fig. 37: Pearson correlation between IC9 (Sig.11+19) and telomere features. Correlations estimated based on 1,254 samples from PCAWG. Telomere features were downloaded from ref[7].

2.13 Sample Level Quality Control and Extraction of Individuals of European Ancestry from Common Germline Variants

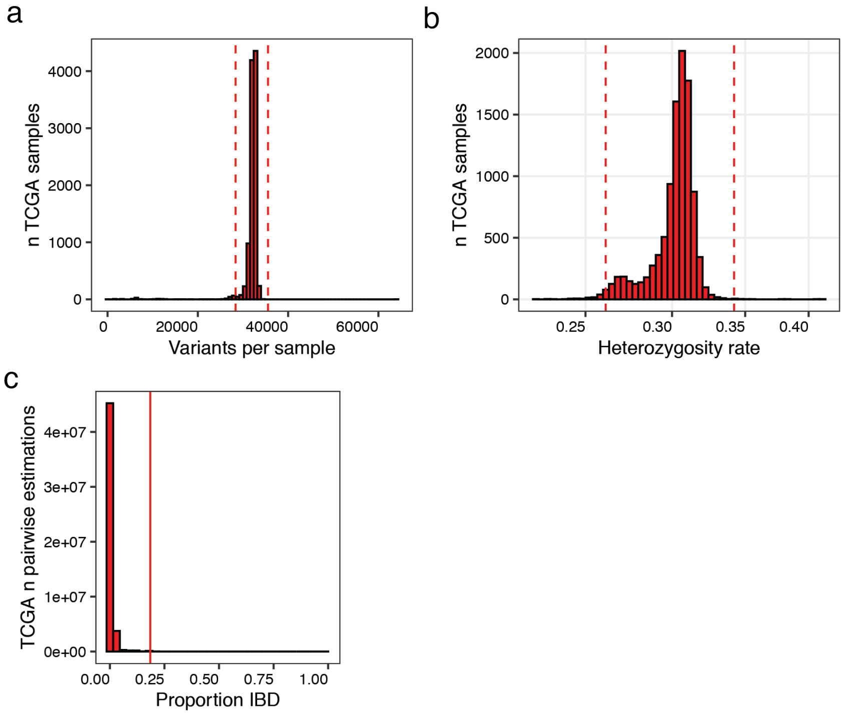

Supplementary Fig. 38: Identification of individuals with outlying total number of variants, outlying heterozygosity rate or high relatedness in TCGA-WES. a, Distribution of total number of variants across samples. Red dashed lines at 1.5 standard deviations away from the mean. **b**, Distribution of heterozygosity rate across samples. Red dashed lines at 3 standard deviations away from the mean. **c**, Proportion of identity-by-descent (IBD) across all sample pairs. Solid red line at 0.185.

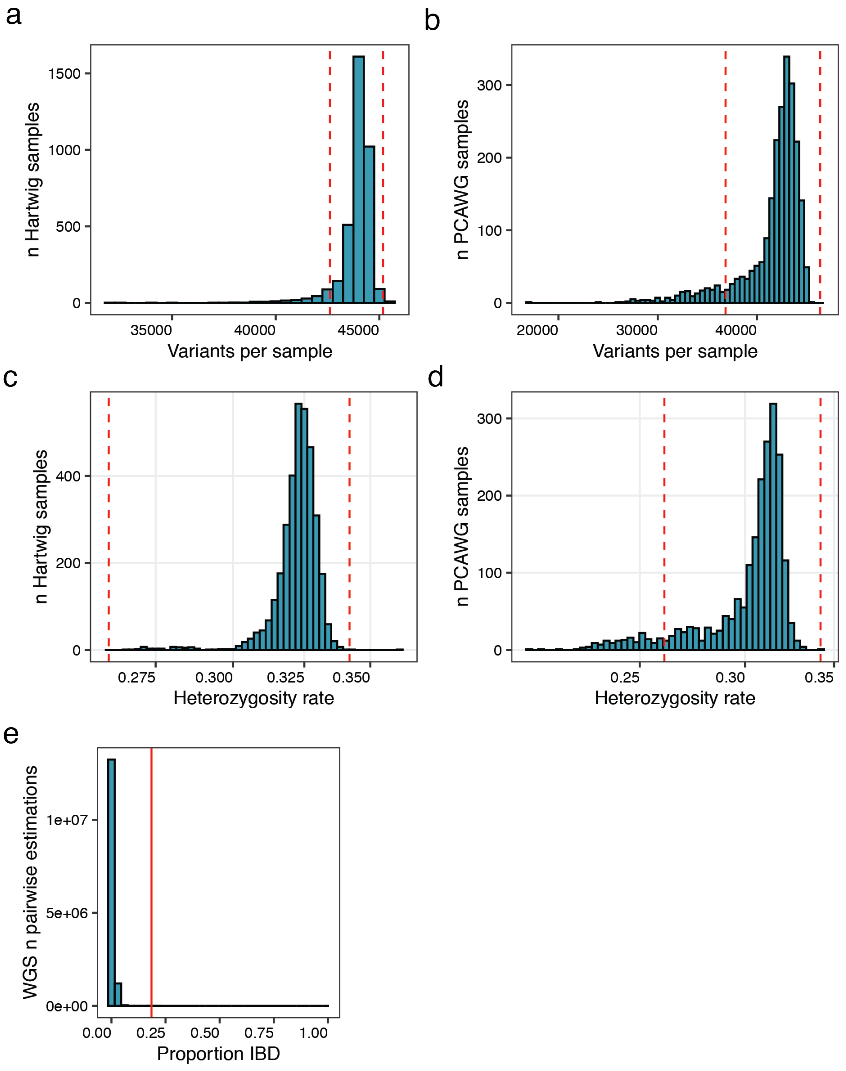

Supplementary Fig. 39: Identification of individuals with outlying total number of variants, outlying heterozygosity rate or high relatedness in PCAWG_Hartwig -WGS. Distribution of total number of variants across samples in **a**, Hartwig and **b**, PCAWG. Red dashed lines at 1.5 standard deviations away from the mean. Distribution of heterozygosity rate across samples in **c**, Hartwig and **d**, PCAWG. Red dashed lines at 3 standard deviations away from the mean. **e**, Proportion of identity-by-descent (IBD) across all sample pairs (PCAWG and Hartwig merged). Solid red line at 0.185.

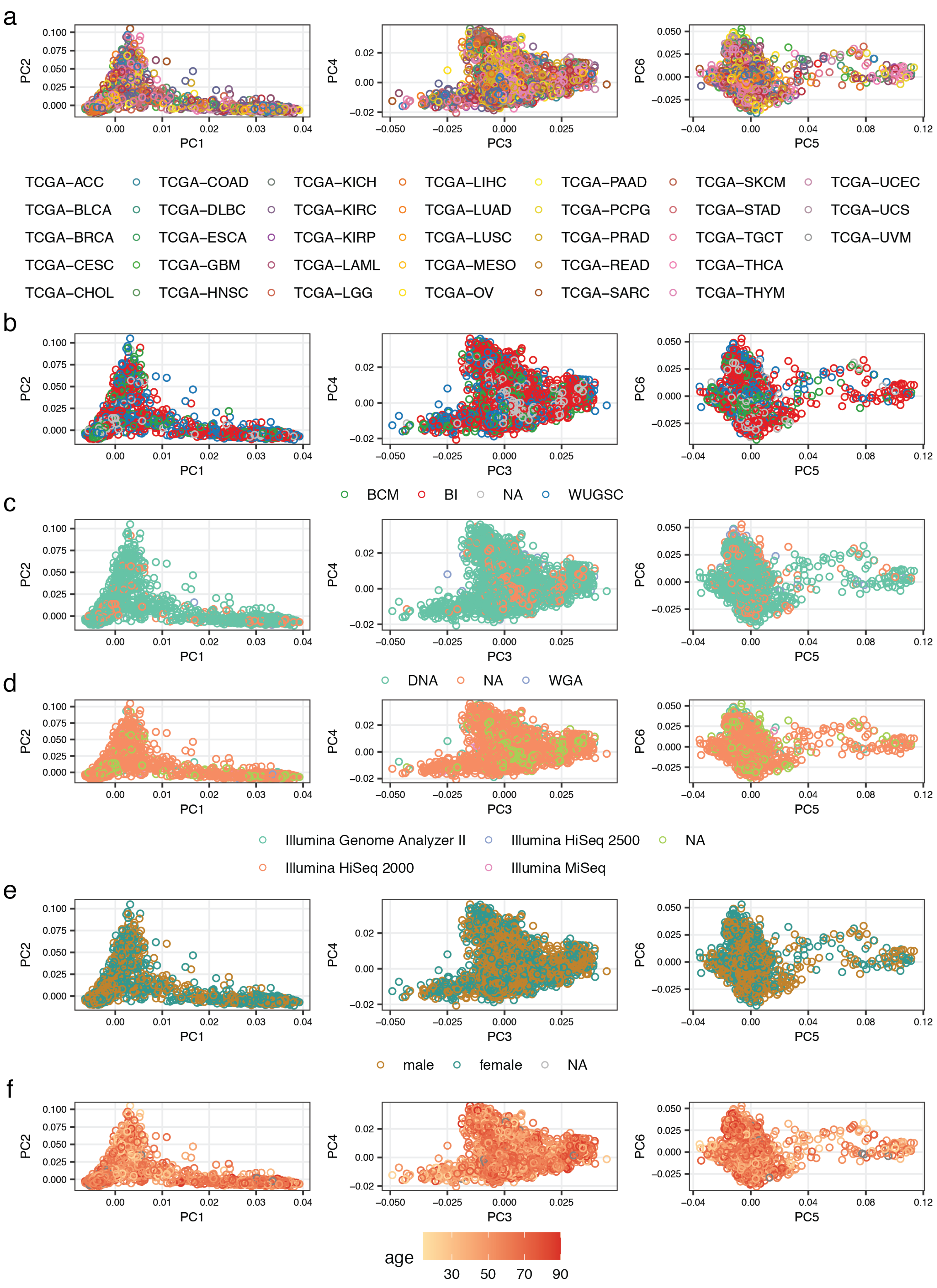

Supplementary Fig. 40: Principal component analysis on common germline variants in TCGA -WES. Principal components 1 to 6 color coded by **a**, TCGA project id, **b**, sequencing center, **c**, whole genome amplification (WGA) status prior to sequencing, **d** , sequencer, **(e)** gender, and **f**, age of diagnosis.

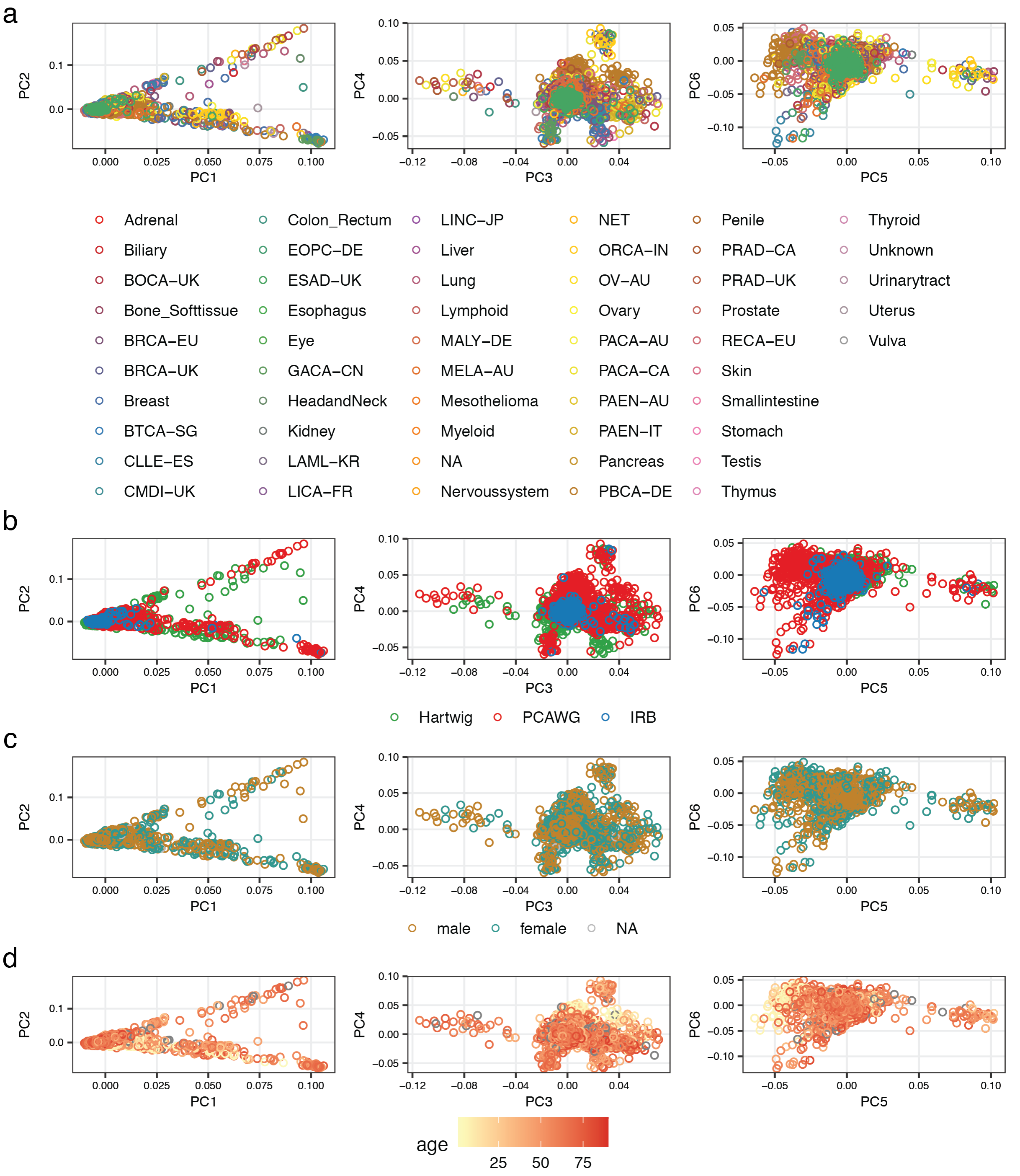

Supplementary Fig. 41: Principal component analysis on common germline variants in PCAWG_Hartwig -WGS. Principal components 1 to 6 color coded by **a**, PCAWG project id or Hartwig tissue of origin, **b**, center/study where germline variants were called, **c**, gender, **d**, age of diagnosis.

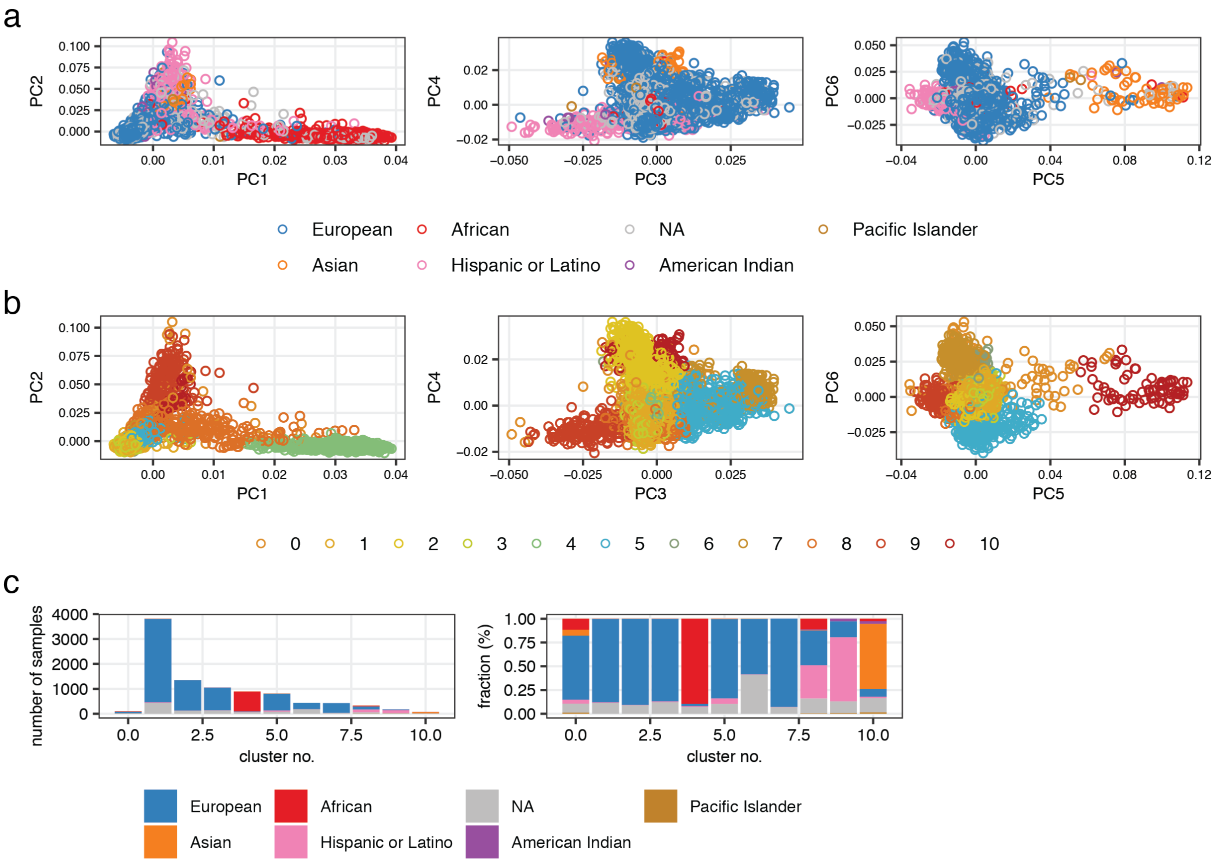

Supplementary Fig. 42: Extraction of European individuals in TCGA -WES. Principal components 1 to 6 color coded by **a**, reported ethnicities and **b**, clustering results using the first 10 principal components. **c**, Overview of clustering results. Samples which could not be assigned to a cluster (cluster no. 0) were excluded.

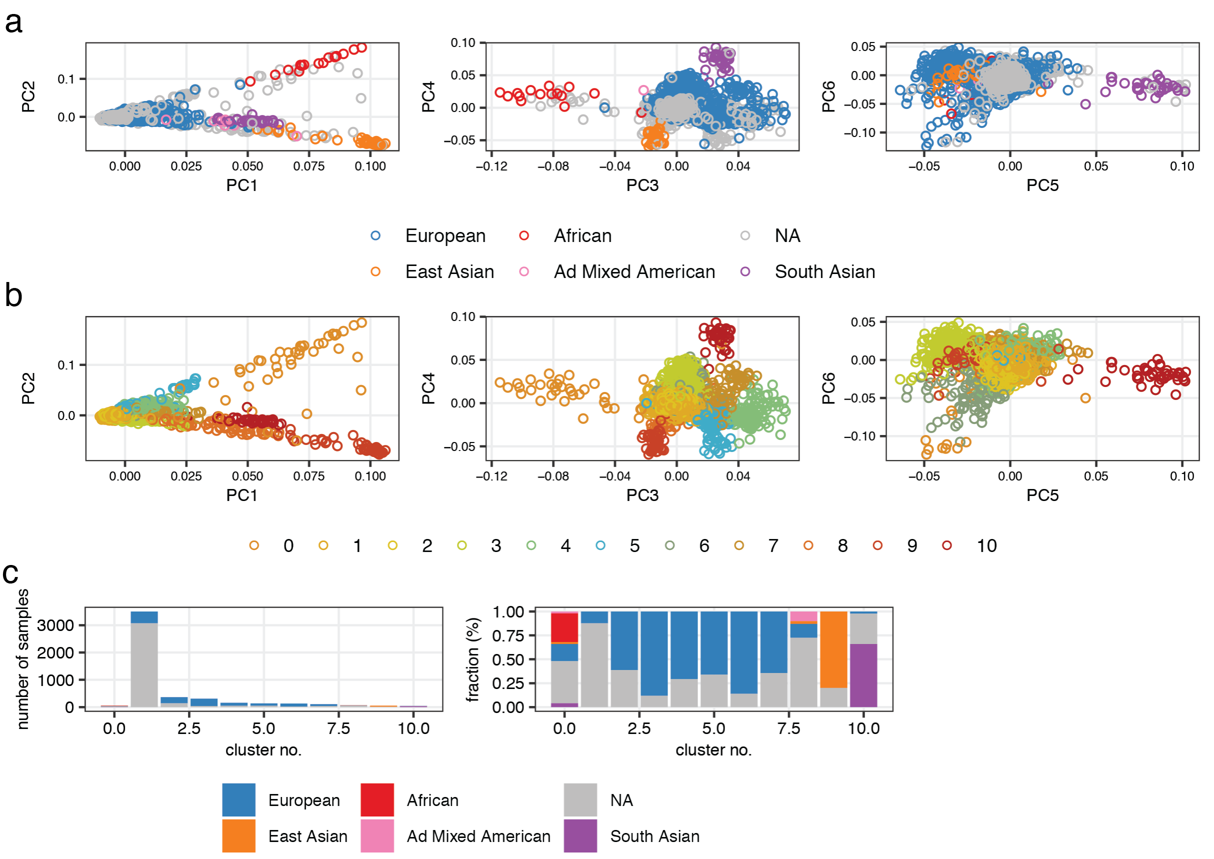

Supplementary Fig. 43: Extraction of European individuals in PCAWG_Hartwig-WGS. Principal components 1 to 6 color coded by **a**, reported ethnicities and **b**, clustering results using the first 10 principal components. **c**, Overview of clustering results. Samples which could not be assigned to a cluster (cluster no. 0) were excluded.

2.14 Comparison Between WES and WGS Extracted Somatic Features

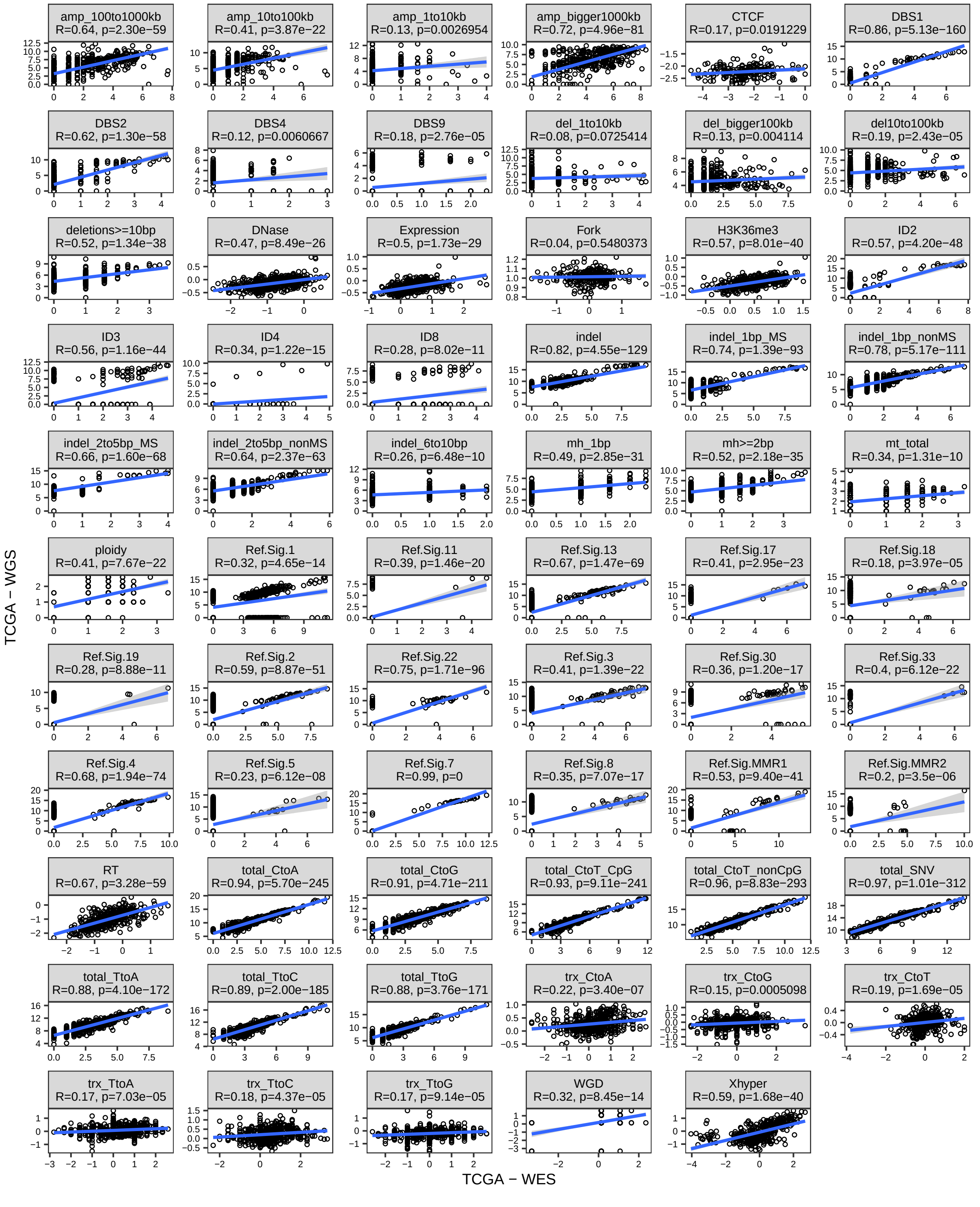

Supplementary Fig. 44: Comparison between WES and WGS extracted somatic features in TCGA. Somatic features were extracted from around 530 individuals from TCGA for which WES as well as WGS data was available. Somatic feature measured in TCGA-WES on the x-axis and in TCGA-WGS on the y-axis. One plot for each somatic features with Pearson correlation and associated p-value from a test for association between paired samples two-sided. Fork: replicative strand bias, RT: replication timing, trx: transcription strand bias, Xhyper: Chromosome X hypermutation.

3 Supplementary Tables

3.1 Replicates Genes - Minimal Distances

Supplementary Table 1: Closest distance between newly replicated genes at a FDR of 2 % and known dMMR/dHR genes. Showing the minimal distance on the respective chromosome between each of the 81 newly replicated genes at a FDR of 2 % and known dMMR (*MSH2*, *MSH6*, and *MLH1*) and dHR (*BRCA1*, *BRCA2*, *PALB2*, and *RAD51C*) genes. Closest distance was calculated using the distance between transcript start and end using RefSeq annotation hg38.

Gene	Closest Gene	Distance in Megabases	Gene	Closest Gene	Distance in Megabases
ACTL6A	*MLH1*	142.51	*NCAPG2*	*PMS2*	152.62
ANKRD28	*MLH1*	21.13	*NFRKB*	none	-
APC	none	-	*NUDT7*	*PALB2*	54.08
APEX1	none	-	*PADI4*	none	-
AQR	none	-	*PARP3*	*MLH1*	14.89
ASCC2	none	-	*PAXIP1*	*PMS2*	148.93
ASCC3	none	-	*PER1*	*BRCA1*	34.89
ATR	*MLH1*	105.4	*PHF8*	none	-
AXIN2	*RAD51C*	6.79	*PIAS1*	none	-
CCNA1	*BRCA2*	4.03	*PIF1*	none	-
CHD3	*BRCA1*	35.13	*PIK3C2B*	none	-
COL7A1	*MLH1*	11.51	*POT1*	*PMS2*	118.81
DIS3L2	*MSH6*	184.15	*PRMT7*	*PALB2*	44.67
DNMT1	none	-	*PRPF19*	none	-
DOCK8	none	-	*RAD51*	none	-
ELP2	none	-	*RBBP5*	none	-
EP300	none	-	*RBBP6*	*PALB2*	0.9
EXO1	none	-	*RBBP8*	none	-
EYA2	none	-	*RECQL*	none	-
FANCC	none	-	*REV3L*	none	-
FANCM	none	-	*RIF1*	*MSH6*	103.6
HERC2	none	-	*SETD1A*	*PALB2*	7.32
HMG20B	none	-	*SETD2*	*MLH1*	9.97
JADE2	none	-	*SETX*	none	-
KANSL1	*BRCA1*	2.9	*SMARCAL1*	*MSH6*	168.61
KANSL3	*MSH6*	48.79	*SMC1A*	none	-
KDM1A	none	-	*SMC1B*	none	-
KDM6A	none	-	*SMC2*	none	-
KDM6B	*BRCA1*	35.19	*SOS1*	*MSH2*	8.28
KMT2B	none	-	*SUPT20H*	*BRCA2*	4.61
KMT2D	none	-	*TELO2*	*PALB2*	22.09
KMT2E	*PMS2*	98.93	*TIMELESS*	none	-
KMT5A	none	-	*TOP2A*	*BRCA1*	2.63
MAD2L2	none	-	*TOP3B*	none	-
MDN1	none	-	*TP53BP1*	none	-
MLH3	none	-	*TRRAP*	*PMS2*	92.87
MSH3	none	-	*TTI2*	none	-
MSL2	*MLH1*	99.1	*VPS72*	none	-
MTOR	none	-	*WRN*	none	-
MUS81	none	-	*ZRANB3*	*MSH6*	87.39
NCAPD2	none	-			

3.2 Genomic Regions

Supplementary Table 2: Covered Genomic Regions with WES and WGS Masks. Estimated lengths of different genomic regions in megabases after applying CRG75 alignability mask on WES and WGS data respectively.

Region	Bin	Size in WES in Megabases	Size in WGS in Megabases
RT	1of6 (late)	1.62	345
	2of6	4.03	382
	3of6	7.94	381
	4of6	12.0	379
	5of6	16.7	373
	6of6 (early)	29.2	356
H3K36me3	0of5 (no marks)	71.0	3,705
	1of5	6.63	143
	2of5	8.54	143
	3of5	11.3	144
	4of5	16.1	147
	5of5 (high density of marks)	29.4	152
Expression	0of5 (no expression)	7.54	2,050
	1of5	14.9	374
	2of5	17.0	425
	3of5	24.2	523
	4of5	34.9	531
	5of5 (high expression)	44.4	531
DNase I	0of5 (no marks)	95.7	3,758
	1of5	6.89	137
	2of5	7.68	136
	3of5	8.85	135
	4of5	9.95	134
	5of5 (high density of marks)	9.95	134
CTCF/cohesin	flanking site $\pm$500 bp	7.04	83.2
	binding site	1.56	17.38
Fork polarity	1of10 (lagging strand)	7.71	199
	2of10	7.65	196
	9of10	6.75	195
	10of10 (leading strand)	5.54	200

3.3 Somatic Components

Supplementary Table 3: Somatic Component Names.

Component	Name
IC1	Sig.17
IC2	Sig.MMR2+ampli.
IC3	dMMR$_{ICA}$
IC4	dHR$_{ICA}$
IC5	Deletions$_{ICA}$
IC6	APOBEC$_{ICA}$
IC7	Sig.18
IC8	Ploidy
IC9	Sig.11+19
IC10	DBS2
IC11	Sig.5$_{ICA}$
IC12	Smoking$_{ICA}$
IC13	Small indels 2bp
IC14	UV$_{ICA}$
IC15	Sig.8
VAE_1	APOBEC$_{VAE2}$
VAE_2	Deletions$_{VAE}$
VAE_3	Sig.5$_{VAE}$
VAE_4	Sig.1
VAE_5	UV$_{VAE}$
VAE_6	dHR$_{VAE1}$
VAE_7	Mitochondria
VAE_8	dHR$_{VAE2}$
VAE_9	Smoking$_{VAE}$
VAE_10	dMMR$_{VAE2}$
VAE_11	APOBEC$_{VAE1}$
VAE_12	X-hypermutation
VAE_13	dMMR$_{VAE1}$
VAE_14	Amplifications

3.4 Cancer Types

Supplementary Table 4: TCGA Study Abbreviations. `https://gdc.cancer.gov/resources-tcga-users/tcga-code-tables/tcga-study-abbreviations`. List of studies, which were ultimately used in association testing (after filtering steps).

Study Abbreviation	Study Name
ACC	Adrenocortical carcinoma
BLCA	Bladder Urothelial Carcinoma
BRCA	Breast invasive carcinoma
CESC	Cervical squamous cell carcinoma and endocervical adenocarcinoma
CHOL	Cholangiocarcinoma
COAD	Colon adenocarcinoma
DLBC	Lymphoid Neoplasm Diffuse Large B-cell Lymphoma
ESCA	Esophageal carcinoma
GBM	Glioblastoma multiforme
HNSC	Head and Neck squamous cell carcinoma
KICH	Kidney Chromophobe
KIRC	Kidney renal clear cell carcinoma
KIRP	Kidney renal papillary cell carcinoma
LAML	Acute Myeloid Leukemia
LGG	Brain Lower Grade Glioma
LIHC	Liver hepatocellular carcinoma
LUAD	Lung adenocarcinoma
LUSC	Lung squamous cell carcinoma
MESO	Mesothelioma
OV	Ovarian serous cystadenocarcinoma
PAAD	Pancreatic adenocarcinoma
PCPG	Pheochromocytoma and Paraganglioma
PRAD	Prostate adenocarcinoma
READ	Rectum adenocarcinoma
SARC	Sarcoma
SKCM	Skin Cutaneous Melanoma
STAD	Stomach adenocarcinoma
THCA	Thyroid carcinoma
THYM	Thymoma
UCEC	Uterine Corpus Endometrial Carcinoma
UCS	Uterine Carcinosarcoma
UVM	Uveal Melanoma

Supplementary Table 5: PCAWG Study Abbreviations. `https://dcc.icgc.org/projects/details`. List of studies, which were ultimately used in association testing (after filtering steps).

Study Abbreviation	Study Name
BOCA-UK	Bone Cancer - UK
BRCA-EU	Breast ER+ and HER2- Cancer - EU/UK
BRCA-UK	Breast Triple Negative/Lobular Cancer - UK
BTCA-SG	Biliary Tract Cancer - SG
CLLE-ES	Chronic Lymphocytic Leukemia - ES
CMDI-UK	Chronic Myeloid Disorders - UK
EOPC-DE	Early Onset Prostate Cancer - DE
ESAD-UK	Esophageal Adenocarcinoma - UK
LICA-FR	Liver Cancer - FR
MALY-DE	Malignant Lymphoma - DE
MELA-AU	Skin Cancer - AU
OV-AU	Ovarian Cancer - AU
PACA-AU	Pancreatic Cancer - AU
PACA-CA	Pancreatic Cancer - CA
PAEN-AU	Pancreatic Cancer Endocrine neoplasms - AU
PAEN-IT	Pancreatic Endocrine Neoplasms - IT
PBCA-DE	Pediatric Brain Cancer - DE
PRAD-CA	Prostate Adenocarcinoma - CA
PRAD-UK	Prostate Adenocarcinoma - UK
RECA-EU	Renal Cell Cancer - EU/FR

Supplementary Table 6: Cancer Type Names. Cancer type names used in this study and respective cancer types from TCGA, PCAWG, and Hartwig which were assigned to it. EAC: oesophageal adenocarcinoma.

Cancer Type Name	Discovery	Validation	
	TCGA Cancer Type	PCAWG Project ID(s)	Hartwig Cancer Type(s)
Bladder	BLCA	-	Urinarytract
Brain_glioma_low	LGG	PBCA-DE	Nervoussystem_Gliomas or _NA
Brain_glioma_multi	GBM	PBCA-DE	Nervoussystem_Gliomas or _NA
Breast	BRCA	BRCA-EU, BRCA-UK	Breast
Colon_Rectum	COAD, READ	-	Colon_Rectum
Kidney	KIRC, KIRP	RECA-EU	Kidney
Lung_ad	LUAD	-	Lung
Lung_sq	LUSC	-	Lung
Ovary	OV	OV-AU	Ovary
Prostate	PRAD	PRAD-CA, PRAD-UK	Prostate
Skin	SKCM	MELA-AU_Cutaneous	Skin_Melanoma or _NA
Stomach_Eso	STAD, ESCA (EAC only[13])	GACA-CN, ESAD-UK	Stomach, Esophagus

Supplementary Table 7: Overview of sample sizes. Corresponding cancer types for the cancer type names can be found in Table 6.

Cancer Type Name	Discovery cohort sample size	Validation cohort sample size
Bladder	323	87
Brain_glioma_low	405	283
Brain_glioma_multi	253	283
Breast	684	656
Colon_Rectum	410	417
Kidney	445	168
Lung_ad	434	299
Lung_sq	373	299
Ovary	199	180
Prostate	386	443
Skin	403	370
Stomach_Eso	363	431
Pancan	6,799	4,683

Supplementary Table 8: Matching GTEx tissues with cancer types in TCGA for proportion expressed across transcripts (PEXT) metric[14]. List showing with which GTEx tissue each cancer type was matched with. Matching was mostly guided by a previous study[15]. When no matching GTEx tissue was identified, mean proportion value from PEXT was used.

TCGA cancer type	Matching GTEx tissue
ACC	AdrenalGland
BLCA	Bladder
BRCA	Breast_MammaryTissue
CESC	Vagina
CHOL	mean_proportion
COAD	Colon_Transverse
DLBC	WholeBlood
ESCA	Esophagus_Mucosa
GBM	Brain_Substantianigra
HNSC	mean_proportion
KICH	Kidney_Cortex
KIRP	Kidney_Cortex
KIRC	Kidney_Cortex
LAML	WholeBlood
LGG	Brain_Amygdala
LIHC	Liver
LUAD	Lung
LUSC	Lung
MESO	mean_proportion
OV	Ovary
PAAD	Pancreas
PCPG	Pituitary
PRAD	Prostate
READ	mean_proportion
SARC	mean_proportion
SKCM	Skin_SunExposed_Lowerleg
STAD	Stomach
THCA	Thyroid
THYM	mean_proportion
UCEC	FallopianTube
UCS	Cervix_Endocervix
UVM	mean_proportion

Supplementary Table 9: Matching GTEx tissues with cancer types in PCAWG for proportion expressed across transcripts (PEXT) metric[14]. List showing with which GTEx tissue each cancer type was matched with. When no matching GTEx tissue was identified, mean proportion value from PEXT was used.

PCAWG cancer type	Matching GTEx tissue
BOCA-UK	mean_proportion
BRCA-EU	Breast_MammaryTissue
BRCA-UK	Breast_MammaryTissue
BTCA-SG	mean_proportion
CLLE-ES	WholeBlood
CMDI-UK	WholeBlood
EOPC-DE	Prostate
ESAD-UK	Esophagus_Mucosa
GACA-CN	Stomach
LAML-KR	WholeBlood
LICA-FR	Liver
LINC-JP	Liver
LIRI-JP	Liver
MALY-DE	WholeBlood
MELA-AU	Skin_SunExposed_Lowerleg
ORCA-IN	mean_proportion
OV-AU	Ovary
PACA-AU	Pancreas
PACA-CA	Pancreas
PAEN-AU	Pancreas
PAEN-IT	Pancreas
PBCA-DE	mean_proportion
PRAD-CA	Prostate
PRAD-UK	Prostate
RECA-AU	Kidney_Cortex

Supplementary Table 10: Matching GTEx tissues with cancer types in Hartwig for proportion expressed across transcripts (PEXT) metric[14]. List showing with which GTEx tissue each cancer type was matched with. When no matching GTEx tissue was identified, mean proportion value from PEXT was used.

Hartwig cancer type	Matching GTEx tissue
Adrenal	AdrenalGland
Biliary	mean_proportion
Bone_Softtissue	mean_proportion
Breast	Breast_MammaryTissue
Colon_Rectum	Colon_Transverse
Doubleprimary	mean_proportion
Esophagus	Esophagus_Mucosa
Eye	mean_proportion
HeadandNeck	mean_proportion
Kidney	Kidney_Cortex
Liver	Liver
Lung	Lung
Lymphoid	WholeBlood
Mesothelioma	mean_proportion
Myeloid	WholeBlood
Nervoussystem	mean_proportion
NET	mean_proportion
Ovary	Ovary
Pancreas	Pancreas
Penile	mean_proportion
Prostate	Prostate
Skin	Skin_SunExposed_Lowerleg
Smallintestine	SmallIntestine_TerminalIleum
Stomach	Stomach
Testis	Testis
Thymus	mean_proportion
Thyroid	Thyroid
Unknown	mean_proportion
Urinarytract	Bladder
Uterus	Uterus
Vulva	Vagina

4 Supplementary References

1. Degasperi, A. *et al.* A practical framework and online tool for mutational signature analyses show inter-tissue variation and driver dependencies. *Nat Cancer* **1**, 249-263 (2020).

2. Alexandrov, L.B. *et al.* Signatures of mutational processes in human cancer. *Nature* **500**, 415-21 (2013).

3. Kucab, J.E. *et al.* A Compendium of Mutational Signatures of Environmental Agents. *Cell* **177**, 821-836 e16 (2019).

4. Hwang, S. *et al.* HumanNet v2: human gene networks for disease research. *Nucleic Acids Res* **47**, D573-D580 (2019).

5. Wu, Y., Poulos, R.C. & Reddel, R.R. Role of POT1 in Human Cancer. *Cancers (Basel)* **12** (2020).

6. Stinus, S., Paeschke, K. & Chang, M. Telomerase regulation by the Pif1 helicase: a length-dependent effect? *Curr Genet* **64**, 509-513 (2018).

7. Sieverling, L. *et al.* Genomic footprints of activated telomere maintenance mechanisms in cancer. *Nat Commun* **11**, 733 (2020).

8. Li, F. *et al.* The histone mark H3K36me3 regulates human DNA mismatch repair through its interaction with MutSalpha. *Cell* **153**, 590-600 (2013).

9. Huang, Y., Gu, L. & Li, G.M. H3K36me3-mediated mismatch repair preferentially protects actively transcribed genes from mutation. *J Biol Chem* **293**, 7811-7823 (2018).

10. Supek, F. & Lehner, B. Clustered Mutation Signatures Reveal that Error-Prone DNA Repair Targets Mutations to Active Genes. *Cell* **170**, 534-547 e23 (2017).

11. Derkach, A., Zhang, H. & Chatterjee, N. Power Analysis for Genetic Association Test (PAGEANT) provides insights to challenges for rare variant association studies. *Bioinformatics* **34**, 1506-1513 (2018).

12. Karczewski, K.J. *et al.* The mutational constraint spectrum quantified from variation in 141,456 humans. *Nature* **581**, 434-443 (2020).

13. Cancer Genome Atlas Research, N. *et al.* Integrated genomic characterization of oesophageal carcinoma. *Nature* **541**, 169-175 (2017).

14. Cummings, B.B. *et al.* Transcript expression-aware annotation improves rare variant interpretation. *Nature* **581**, 452-458 (2020).

15. Zeng, W.Z.D., Glicksberg, B.S., Li, Y. & Chen, B. Selecting precise reference normal tissue samples for cancer research using a deep learning approach. *BMC Med Genomics* **12**, 21 (2019).
