# Supplementary material for: The impact of rare germline variants on human somatic mutation processes
Source: Nat Commun. 2022 Jun 28;13:3724. doi: 10.1038/s41467-022-31483-1 (PMC9240060; doi:10.1038/s41467-022-31483-1)
Supplement: Supplementary file 3 — Description to Additional Supplementary Information [file 41467_2022_31483_MOESM3_ESM.pdf]

Description of Additional Supplementary Files

Supplementary Text, Figures and Tables.

Supplementary Dataset 1: List of 891 selected genes used for testing in this study.

Supplementary Dataset 2: List of genes replicating at a FDR of 1 %.

Supplementary Dataset 3: List of genes replicating at a FDR of 2 %.
