# Supplementary material for: The impact of rare germline variants on human somatic mutation processes
Source: Nat Commun. 2022 Jun 28;13:3724. doi: 10.1038/s41467-022-31483-1 (PMC9240060; doi:10.1038/s41467-022-31483-1)
Supplement: Supplementary file 5 — Reporting Summary [file 41467_2022_31483_MOESM5_ESM.pdf]

nature portfolio

Corresponding author(s): Lehner, Supek

Last updated by author(s): Jun 5, 2022

Reporting Summary

Nature Portfolio wishes to improve the reproducibility of the work that we publish. This form provides structure for consistency and transparency in reporting. For further information on Nature Portfolio policies, see our Editorial Policies and the Editorial Policy Checklist.

Statistics

For all statistical analyses, confirm that the following items are present in the figure legend, table legend, main text, or Methods section.

n/a	Confirmed	
☐	☒	The exact sample size (*n*) for each experimental group/condition, given as a discrete number and unit of measurement
☒	☐	A statement on whether measurements were taken from distinct samples or whether the same sample was measured repeatedly
☐	☒	The statistical test(s) used AND whether they are one- or two-sided<br>*Only common tests should be described solely by name; describe more complex techniques in the Methods section.*
☐	☒	A description of all covariates tested
☐	☒	A description of any assumptions or corrections, such as tests of normality and adjustment for multiple comparisons
☐	☒	A full description of the statistical parameters including central tendency (e.g. means) or other basic estimates (e.g. regression coefficient) AND variation (e.g. standard deviation) or associated estimates of uncertainty (e.g. confidence intervals)
☐	☒	For null hypothesis testing, the test statistic (e.g. *F*, *t*, *r*) with confidence intervals, effect sizes, degrees of freedom and *P* value noted<br>*Give P values as exact values whenever suitable.*
☒	☐	For Bayesian analysis, information on the choice of priors and Markov chain Monte Carlo settings
☒	☐	For hierarchical and complex designs, identification of the appropriate level for tests and full reporting of outcomes
☐	☒	Estimates of effect sizes (e.g. Cohen's *d*, Pearson's *r*), indicating how they were calculated

Our web collection on statistics for biologists contains articles on many of the points above.

Software and code

Policy information about availability of computer code

Data collection	No special software was used for data collection; we used previously collected data available from either public databases or other publications as described in the Methods and Data availability statement.
Data analysis	Analysis was performed in R (3.5.0) with R packages fastICA (1.2-1), cluster (2.0.6), SKAT (2.0.1), fastDummies (1.6.3), MASS (7.3_53.1), tclust (1.4-2), and visualization using R packages ggplot (3.3.5), ggrepel (0.9.1) and corrplot (0.9). Variational autoencoder was run in a Singularity container using Python (3.6.9), keras (2.2.4) and tensorflow (1.15.5). Extraction of somatic mutations and signatures with python tool (python version 3.8) SigProfilerMatrixGenerator version 1.1.26 (https://github.com/AlexandrovLab/SigProfilerMatrixGenerator) and SigProfilerExtractor version 1.1.0 (https://github.com/AlexandrovLab/SigProfilerExtractor). Organ-specific signatures were extracted in R with signature.tools.lib package from https://github.com/Nik-Zainal-Group/signature.tools.lib. Genetic variants were annotated with ANNOVAR (version 2019-10-24). Common variants were processed via PLINKv1.90b6.1. Processing of genomic regions with bedtools 2.27. Power analysis with R tool PAGEANT (https://github.com/andrewhaoyu/PAGEANT). Code for custom scripts available at https://github.com/lehner-lab/RDGVassociation

For manuscripts utilizing custom algorithms or software that are central to the research but not yet described in published literature, software must be made available to editors and reviewers. We strongly encourage code deposition in a community repository (e.g. GitHub). See the Nature Portfolio guidelines for submitting code & software for further information.

Data

Policy information about availability of data

All manuscripts must include a data availability statement. This statement should provide the following information, where applicable:

- Accession codes, unique identifiers, or web links for publicly available datasets
- A description of any restrictions on data availability
- For clinical datasets or third party data, please ensure that the statement adheres to our policy

In this study, published datasets were reanalyzed. TCGA WES bam files of primary tumors and matched normal samples (dbGaP accession ID phs000178 [https://www.ncbi.nlm.nih.gov/projects/gap/cgi-bin/study.cgi?study_id=phs000178.v11.p8], restricted access that can be applied to following instructions on dbGaP) were downloaded from the TCGA repository at NCI Genomic Data Commons [https://portal.gdc.cancer.gov/]. Somatic mutation calls for TCGA were downloaded from the MC3 project (mc3.v0.2.8.PUBLIC.maf.gz in [https://gdc.cancer.gov/about-data/publications/mc3-2017]). Germline and somatic calls from PCAWG excluding ESAD-UK and MELA-AU were downloaded from the ICGC data portal [https://dcc.icgc.org/repositories]; these were available under restricted access, which can be applied for via the ICGC DACO [https://daco.icgc-argo.org/]. Bam files for tumor and normal samples from MELA-AU (dataset ID: EGAD00001003388 [https://ega-archive.org/datasets/EGAD00001003388]) and ESAD-AU (dataset ID: EGAD00001003580 [https://ega-archive.org/datasets/EGAD00001003580]) were from the European Genome-Phenome Archive ([https://ega-archive.org]); they are available under restricted access, which can be applied for via the ICGC DACO [https://daco.icgc-argo.org/]. Mitochondrial somatic mutation calls in PCAWG were downloaded from [https://ibl.mdanderson.org/tcma/mutation.html]. Hartwig somatic and germline variant calls were downloaded after acquiring restricted data access from the Hartwig Medical Foundation [https://www.hartwigmedicalfoundation.nl/en/], request number DR-069; requests can be submitted at [https://www.hartwigmedicalfoundation.nl/en/data/data-acces-request/]. Replication timing data from lymphoblastoid cell lines to calculate the replicative strand bias was downloaded from [http://mccarrolllab.org/resources/]. Processed genomic region densities of expression, histone mark H3K36me3, replication timing, CTCF/cohesin binding sites, and DNase I hypersensitive sites were obtained by contacting authors of original publication [https://doi.org/10.1016/j.cell.2017.07.003]. Genomic regions for the CRG75 alignability track and blacklisted regions by Duke and DAC were obtained from the UCSC Genome Browser [https://genome.ucsc.edu/cgi-bin/hgTables]. GnomAD v2 allele frequencies and pext scores were obtained from the gnomAD browser [https://gnomad.broadinstitute.org/downloads]. Gene coordinates were obtained from the UCSC genome browser [https://genome.ucsc.edu/cgi-bin/hgTables]. Regions with high amount of linkage disequilibrium were downloaded from [https://github.com/meyer-lab-cshl/plinkQC/tree/master/inst/extdata]. Pre-computed SpliceAI scores were downloaded from Illumina Basespace [https://basespace.illumina.com/projects/66029966]. Download of SpliceAI scores are free, but require generation of a free account at Illumina Basespace. Complete list of tested 891 genes in Supplementary Dataset 1. Missense tolerance score annotations were obtained from [http://biosig.unimelb.edu.au/mtr-viewer/downloads] and constrained coding region annotations from [http://quinlanlab.org/blog/2018/12/20/constrained-coding-regions.html]. Interaction scores from STRING v11.5 were downloaded from [https://string-db.org/cgi/download?sessionId=bPz0GBvgDw3p] and scores from HumanNet v3 (HumanNet-FN) from [https://www.inetbio.org/humannet/download.php]. Source data are provided with this paper.

Field-specific reporting

Please select the one below that is the best fit for your research. If you are not sure, read the appropriate sections before making your selection.

☒ Life sciences ☐ Behavioural & social sciences ☐ Ecological, evolutionary & environmental sciences

For a reference copy of the document with all sections, see nature.com/documents/nr-reporting-summary-flat.pdf

Life sciences study design

All studies must disclose on these points even when the disclosure is negative.

Sample size	Sample sizes were not predetermined since the goal was to have as many samples as possible. Thus, all samples from TCGA, PCAWG, and Hartwig were utilized. After filtering steps (see Data exclusions), the discovery cohort TCGA-WES involved samples from 6,799 individuals for association testing, and the discovery cohort PCAWG-WGS + Hartwig-WGS involved samples from 4,683 individuals. Discovery and validation cohort were independent without any overlap of individuals. Sample sizes of individual cancer types can be found in Supplementary Table 7.
Data exclusions	In TCGA 93 samples which were reported to be positive for human papillomaviruses in head and neck cancer samples (https://doi.org/10.1158/2159-8290.cd-17-1018) were excluded to prevent that this mechanism confounds the analysis. Based on the common variants analysis duplicated and/or related individuals were removed to not bias the analysis (1,021 individuals). Samples with a high deviation in the number of common variants were removed following established data qulity guidelines (630 individuals) (https://doi.org/10.1038/nprot.2010.116). To exclude the possibility of population-specific germline variants showing spurious associations with mutational processes, only individuals of European ancestry were kept for the analysis since this was the biggest group (12,555 individuals retained). Tumors with a low mutational burden (<10 SNVs, 1073 indidivuals) were excluded as it was performed in the PCAWG study (https://doi.org/10.1038/s41586-020-1969-6).
Replication	Hits were identified in the discovery cohort TCGA-WES. Testing in the discovery cohort was repeated several times while finding optimal hyperparameters and identifying the most optimal test. Testing with SKAT-O led to more hits than with burden testing alone. Testing for replication in the validation cohort PCAWG-WGS + Hartwig-WGS with SKAT-O was performed once.
Randomization	Randomization in association testing was performed for the estimation of empirical False Discovery Rates via two approaches: (1) by randomly shuffling the somatic components matrix and (2) by randomly selecting a list of 1,000 genes and performing the same analysis.<br>Furthermore, randomization was performed in the network analysis. For all network analyses, in each randomization a random set of genes was taken out of the list of 891 genes while controlling for the the total number of interactions (also called node connectivity in this study) each gene has to not confound the analysis (e.g. BRCA1 naturally interacts with a lot of genes). This was achieved, by splitting the genes into 10 equal sized bins based on their total number of interactions. During randomization the total number of interactions was controlled for by randomly selecting the same number of genes from each bin as in the control group.

	Another randomization was performed when comparing the frequency of rare pLoF variants in the cancer cohorts between the replicated genes and a random selection of genes. For each replicated gene, 10 randomly selected length matched genes were selected. It was controlled for length (gene length covered after applying genomic region filter such as CRG75) to remove this bias from the analysis (e.g long genes would have a higher probability to have a rare pLoF variant and vice versa).
Blinding	n/a (this is an observational study, experimental interventions were not performed)

Reporting for specific materials, systems and methods

We require information from authors about some types of materials, experimental systems and methods used in many studies. Here, indicate whether each material, system or method listed is relevant to your study. If you are not sure if a list item applies to your research, read the appropriate section before selecting a response.

Materials & experimental systems

n/a	Involved in the study	
☒	☐	Antibodies
☒	☐	Eukaryotic cell lines
☒	☐	Palaeontology and archaeology
☒	☐	Animals and other organisms
☒	☐	Human research participants
☒	☐	Clinical data
☒	☐	Dual use research of concern

Methods

n/a	Involved in the study	
☒	☐	ChIP-seq
☒	☐	Flow cytometry
☒	☐	MRI-based neuroimaging
